# Supplementary material for: Molecular near-infrared triplet-triplet annihilation upconversion with eigen oxygen immunity
Source: Nat Commun. 2024 Mar 9;15:2157. doi: 10.1038/s41467-024-46541-z (PMC10924867; doi:10.1038/s41467-024-46541-z)
Supplement: Supplementary file 1 — Supplementary Information [file 41467_2024_46541_MOESM1_ESM.pdf]

Supplementary Information

Table of Contents

1. **Supplementary Methods** 2

1.1 Instrumentation 2

1.2 Software 2

1.3 Limited of detection 2

1.4 Evaluation of the encapsulation efficiency 2

1.6 Synthesis of carboxylic-containing Cy7 dye 3

1.7 Synthesis of BTTQD 1-3 4

1.8 Synthesis of F127-NH_2 11

1.9 Preparation of TTA nanomicells with and without Cy 7 11

1.10 Preparation of various ROS/RNS stock solutions 12

2. **Supplementary Discussion** 14

2.1 Kinetic analysis of transient absorption spectra[8-11] 14

2.2 Kinetic analysis from classical steady/transient -state measurements 15

2.3 Reliability assessment of experimentally observed TTET rates 16

2.4 Oxygen quenching efficiency for the triplets of sensitizer and annihilator 17

2.5 Measurement of upconverstion quantum yield (UCQY) 18

2.6 Photoluminescence quantum yield (PLQY) of IR806 and BTTQD 1-3 19

3. **Supplementary Figures** 22

4. **Supplementary Tables** 47

5. **Supplementary References** 52

1. Supplementary Methods

1.1 Instrumentation

The size and morphology of nanoparticles were characterized by transmission electron microscope (TEM, Tecnai G2 Spirit Twin 12). Proton nuclear magnetic resonance (^1H NMR) spectrum was recorded on a Bruker 400 using $CDCl_3$ as solvent at room temperature. Absorption spectra were measured using an Agilent Cary5000 UV-Vis-NIR spectrophotometer, by dissolving substances in solvent in a quartz cuvette with a path length of 1 cm. Upconversion luminescence spectra and decay curves were collected by Edinburgh FLS1000 spectrofluorometer equipped with an 808 nm diode laser (MLL-III-808-2W, Changchun New Industries Optoelectronics Tech Co.) that can operate in both continuous-wave and pulse mode. MALDI-TOF MS analyses were performed in positive reflection mode on a 5800 proteomic analyzer (Applied Biosystems, Framingham, MA, USA) with a YAG:Nd laser. Dynamic light scattering (DLS) was carried out on a Malvern Zetasizer 3600 (Malvern Instruments).

1.2 Software

ImageJ 1.52p (NIH, USA); Origin2019, Adobe illustrator CS6; MestReNova 6.1.0-6224.

1.3 Limited of detection

The limited of detection of TTA-NMs toward $ONOO^-$ was calculated according to equation:

$$LOD = \frac{3\delta}{K} \quad (1)$$

where δ is the standard deviations for five repeated luminescent measurements of blank samples; K is the slope for the upconverted emission intensity versus the concentration of $ONOO^-$.

1.4 Evaluation of the encapsulation efficiency

The encapsulation efficiency was determined by comparing the characteristic absorbance differences of IR806 and BTTQD 2 before and after encapsulation in TTA-

NMs. Initially, the absorbance of IR806 solution (10 μM) and BTTQD 2 solution (100 μM) were determined. Subsequently, the absorbance of the TTA-NMs aqueous solution, obtained after repeated centrifugation and washing, was measured. As the absorbance spectra of TTA-NMs encompass the characteristic absorption bands of both IR806 and BTTQD 2, the encapsulation efficiency of IR806 and BTTQD 2 was estimated by the ratio of characteristic absorbance peak before and after encapsulation in TTA-NMs. The load efficiency of Cy7 onto the TTA-NMs surface can also be estimated using the same method.

1.5 Synthesis of IR806 dye

IR806 was synthesized based on our previous work.[1] Specifically, 4-meracptobenzoic acid (115 mg, 0.75 mmol) and iodide IR780 (250 mg, 0.375 mmol) were dissolved in anhydrous DMF and stirred overnight at room temperature under the protection of an inert gas. After the reaction is complete, the solvent DMF is removed from the reaction mixture using a vacuum pump to dryness. The resulting crude product is then dissolved again in DCM. Following that, an excess of ether is used as a precipitant to allow the product to precipitate from the solution. The precipitate is then collected by filtration to obtain a dark green solid product. The dark green crude products were purified by column chromatography using petroleum CH_3CN/methyl alcohol to afford the corresponding IR806 in 70% yield (204 mg, CH_3CN:CH_3OH = 7:3). NMR (500 MHz, CD_3OD): δ 8.52 (d, 2H), 7,92 (d, 2H), 7.35–7.61 (m, 10H), 6.33 (d, 2H), 4.11 (t, 4H), 2.70 (m, 4H), 1.92 (m, 2H), 1.73 (q, 4H), 1.19 (s, 13H), 0.89 (t, 6H).

1.6 Synthesis of carboxylic-containing Cy7 dye

Carboxylic-containing Cy7 was synthesized using the standard Schlenk technique and following a procedure reported previously with some modifications.[2] A mixture of IR780 iodide (333 mg, 0.5 mmol) and 3-mercaptopropionic acid (85 μL, 1 mmol) were dissolved in 10 mL of anhydrous DMF. Subsequently, Et_3N (137 μL, 1 mmol) was added to the reaction system to deprotonate the 3-mercaptopropionic acid. The reaction was stirred at room temperature overnight under N_2 atmosphere. The solution was

filtered through a 0.45 μm PTFE syringe filter, followed by slowly adding diethyl ether to precipitate the product. The dark green crude products were purified by column chromatography using petroleum DCM/methyl alcohol to afford the corresponding Cy7 in 81% yield (280 mg, CH_2Cl_2: CH_3OH = 100:1 ~ 10:1). ^{1}H NMR (400 MHZ, CD_3OD): δ 8.86 (d, 2H), 7.54 (d, 2H), 7.45-7.33 (m, 2H), 7.19 (dd, 4H), 6.24 (d, 2H), 4.07 (t, 4H), 3.31 (dt, 4H), 3.03 (t, 2H), 2.71 (s, 2H), 2.60 (t, 2H), 1.86 (dd, 4H), 1.71 (s, 12H), 1.06 (t, 6H).

1.7 Synthesis of BTTQD 1-3

Synthesis of compound 3: Compound **3** was synthesized based on the scheme we reported earlier.[3] The synthetic route of the desired products is depicted in Supplementary Figure 1. Dissolve compound **1** (53 mg, 0.1 mmol) and **2** (24 mg, 0.1 mmol) in 5 mL of acetic acid under nitrogen atmosphere, heat the mixture to 100 °C, and stir for 5 hours, the solution gradually turns red from yellow, and the reaction process is monitored by TLC until the raw materials disappear. The mixture is cooled to room temperature and then concentrated under reduced pressure, followed by adding 20 mL of distilled water into the reaction bottle to remove acetic acid as much as possible. The solution is then treated with DCM (3×10 mL), combine the organic phase, and dry with an appropriate amount of Na_2SO_4. The resulting crude products are subsequently purified by column chromatography using petroleum ether/DCM to afford the corresponding compound **3** in 40% yield (23.8 mg, petroleum ether: dichloromethane = 9:1) ^{1}H NMR (400 MHz, Chloroform-d) δ 8.72 (d, J = 2.0 Hz, 1H), 7.80 (dd, J = 8.4, 2.0 Hz, 1H), 7.70 (d, J = 8.4 Hz, 1H), 1.31 (dd, J = 27.2, 5.2 Hz, 42H).

Synthesis of BTTQD 1 and 2: Compound **3** (30 mg, 0.04 mmol) is placed in a single necked bottle with 1,4-benzenediboronic acid bis(pinacol) ester (7 mg, 0.02 mmol), K_2CO_3 (60 mg, 0.4 mmol), and catalyst Pd(dppf)$_2$Cl$_2$ · CH_2Cl_2 (4 mg, 0.004 mmol), and a condenser tube is added. The reaction system is extracted three times under nitrogen atmosphere, and the newly steamed THF (20 mL) solution is added to the single necked bottle, heat and reflux overnight. The reaction process is monitored by TLC, After the reaction is completed, dry the solvent and heat it with 10 mL of

chloroform for dissolution. Add silica gel for stir frying, and purify the crude product (petroleum ether: chloroform = 4:1) by column chromatography to obtain **BTTQD 1** with a yield of 52% (13.4 mg). ^{1}H NMR (400 MHz, Chloroform-d) δ 8.91 (d, J = 1.9 Hz, 2H), 8.07 (dd, J = 8.3, 1.9 Hz, 2H), 7.96 (d, J = 8.3 Hz, 2H), 7.92 (s, 4H), 1.32 (dd, J = 8.3, 4.3 Hz, 84H). MALDI-TOF MS, calculated for $C_{78}H_{94}N_8S_4Si_4$ [m/z] 1382.6, found 1382.6, CCDC No.: 2099068. As for the **BTTQD 2**, the reaction process is similar to **BTTQD 1** using 4,4'-biphenyldiboronic acid dipinacol ester as starting materials.[1] Yield: 56%, 14.8 mg. H NMR (400 MHz, Chloroform-d) δ 8.88 (d, J = 1.8 Hz, 2H), 8.03 (dd, J = 8.3, 1.8 Hz, 2H), 7.93 (d, J = 8.2 Hz, 2H), 7.87 (q, J = 8.2 Hz, 8H), 1.33 (dd, J = 16.4, 4.7 Hz, 84H). MALDI-TOF MS, calculated for $C_{84}H_{98}N_8S_4Si_4$ [m/z] 1458.6, found 1458.6. Due to the poor solubility of these products, the corresponding ^{13}C data could not be obtained.

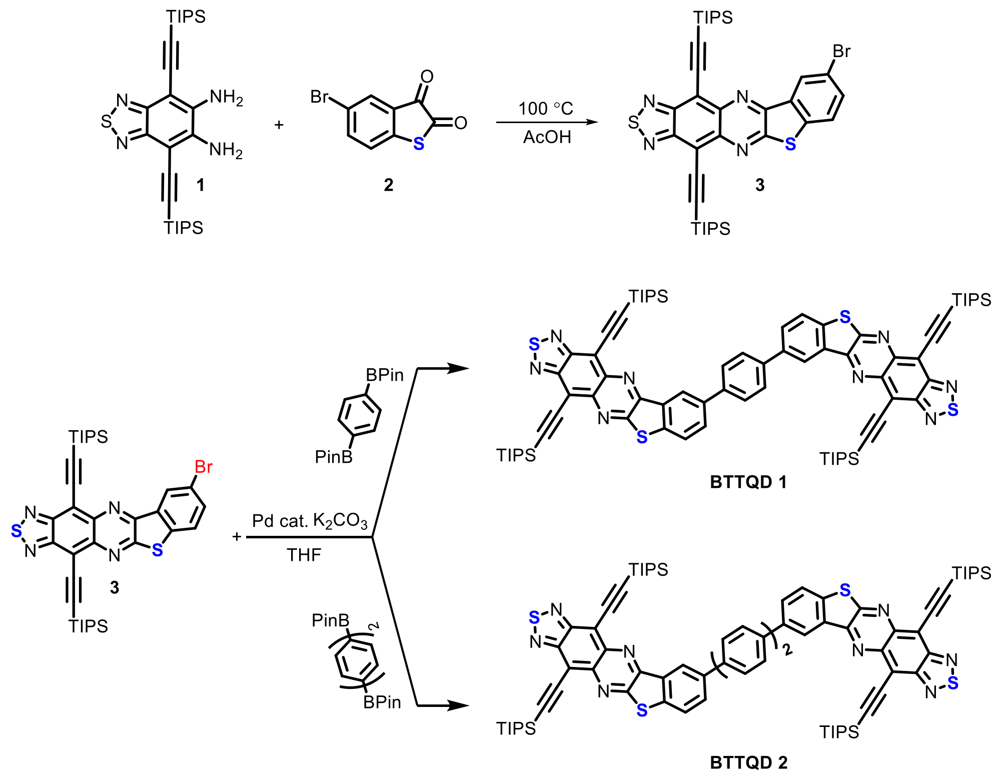

Supplementary Figure 1. Schematic illustration of the synthesis of BTTQD 1 and 2.

Synthesis of compound 5: Compound **5** was synthesized based on the scheme we reported earlier.[4] The synthetic route of the desired products is depicted in

Supplementary Figure 2. Dissolve compound **4** (57 mg, 0.1 mmol) and **2** (24 mg, 0.1 mmol) in 5 mL of acetic acid under nitrogen atmosphere, heat the mixture to 100 °C, and stir for 5 hours, The solution gradually changes from brown to dark brown, and the reaction process is monitored by TLC until the raw materials disappear. The mixture is cooled to room temperature and then concentrated under reduced pressure, followed by adding 20 mL of distilled water into the reaction bottle to remove acetic acid. The solution is then treated with DCM (3×10 mL), combine the organic phase, and dry with an appropriate amount of Na_2SO_4. The resulting crude products are subsequently purified by column chromatography using petroleum ether/DCM to afford the corresponding compound **5** in 42% yield (petroleum ether: DCM = 8:1) ^{1}H NMR (400 MHz, Chloroform-d) δ 9.47-9.34 (m, 2H), 8.73 (d, J = 2.0 Hz, 1H), 8.09 (dd, J = 6.5, 3.2 Hz, 2H), 7.77 (dd, J = 8.4, 2.0 Hz, 1H), 7.69 (d, J = 8.4 Hz, 1H), 7.61-7.50 (m, 2H), 1.37 (dd, J = 28.0, 4.5 Hz, 42H).

Synthesis of BTTQD 3: Compound **5** (39 mg, 0.05 mmol) is placed in a single necked bottle with 4,4'-biphenyldiboronic acid dipinacol ester (9 mg, 0.02 mmol), K_2CO_3 (75 mg, 0.5 mmol), and catalyst Pd(dppf)$_2$Cl$_2$ · CH_2Cl_2 (4 mg, 0.004 mmol), and a condenser tube was added. The reaction system is extracted three times under nitrogen atmosphere, and the bubbled deoxygenated THF/H_2O (20 mL/2 mL) mixed solution Is added to the single necked bottle, heat and reflux overnight. The solution gradually fades from red and insoluble green solid precipitates. TLC monitors the reaction process, and when no raw materials are left, spin dry the solvent and heat it with 10 mL of chloroform for dissolution. Add silica gel to stir fry the sample, and purify the crude product (petroleum ether: chloroform=4:1) by column chromatography to obtain a dark green solid **BTTQD 3** with a yield of 34% (22.1 mg).^{1}H NMR (400 MHz, Chloroform-d) δ 9.45 (d, J = 14.5 Hz, 4H), 8.93 (s, 2H), 8.10 (s, 4H), 8.02 (d, J = 8.6 Hz, 2H), 7.90 (q, J = 10.2, 7.4 Hz, 10H), 7.55 (d, J = 7.8 Hz, 4H), 1.40 (d, J = 19.4 Hz, 84H). MALDI-TOF MS, calculated for $C_{100}H_{110}N_4S_2Si_4$ $[M+H]^+$ 1543.7, found 1543.7. Due to the poor solubility of these products, the corresponding ^{13}C data could not be obtained.

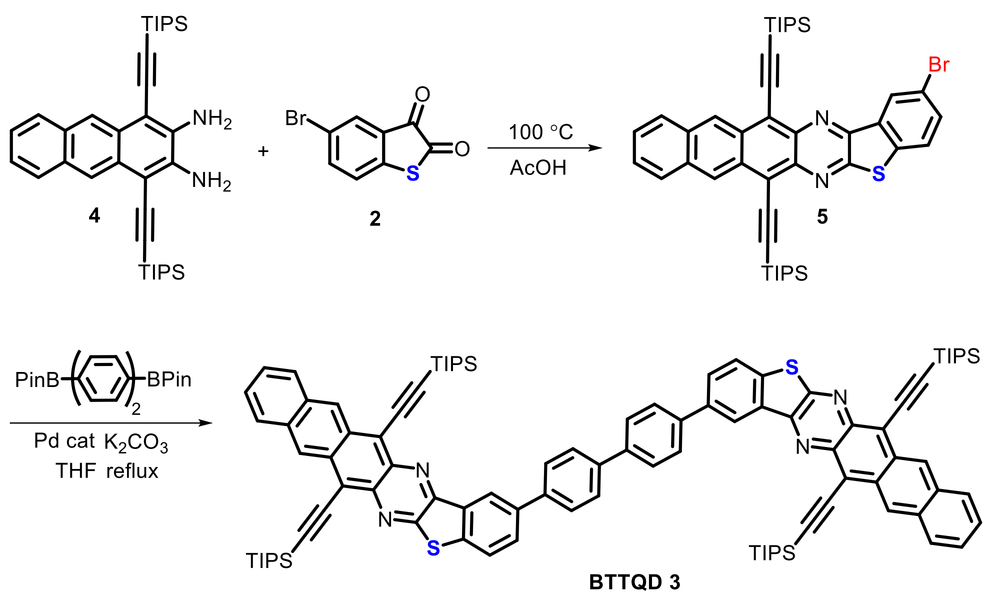

Supplementary Figure 2. Schematic illustration of the synthesis of BTTQD 3.

NMR and MS spectra

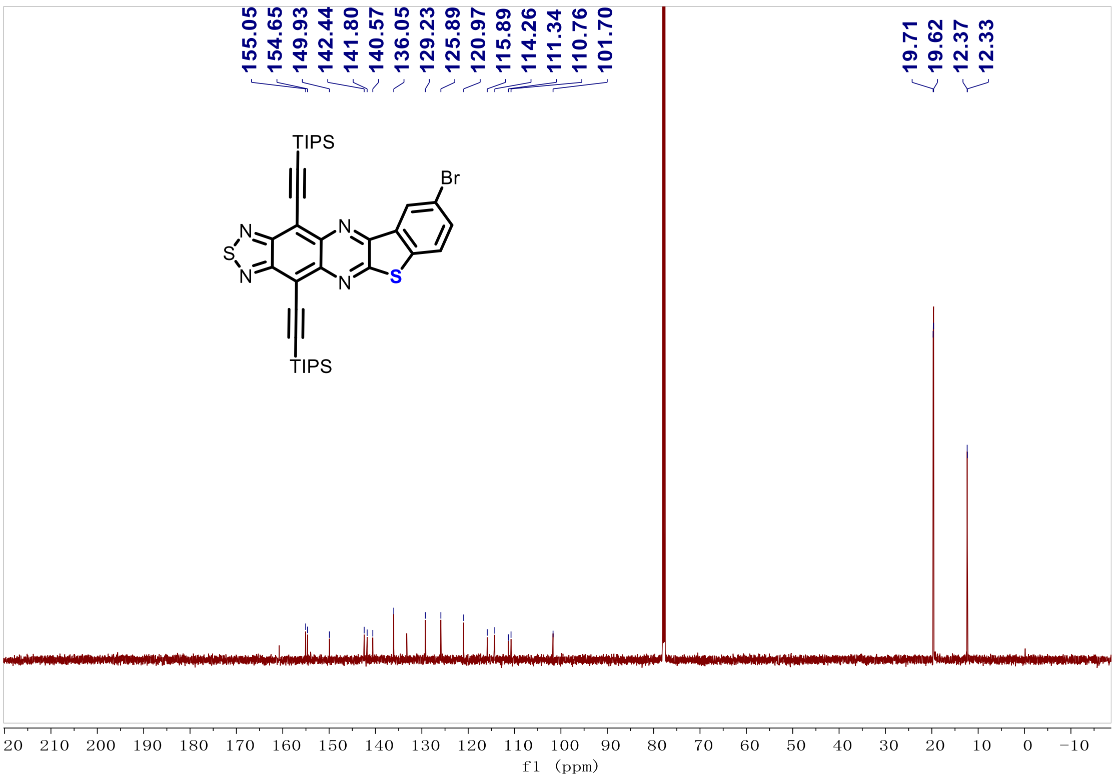

Supplementary Figure 3. ^{1}H-NMR of compound **3**.

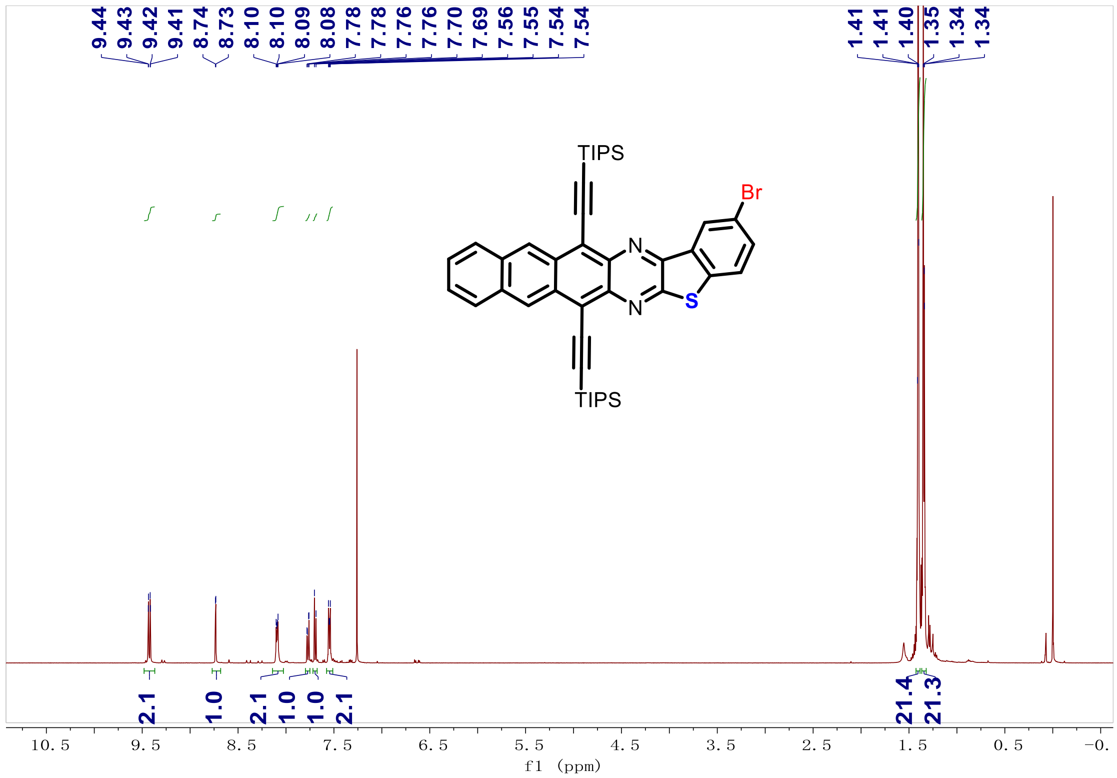

Supplementary Figure 4. ^{1}H-NMR of compound **5**.

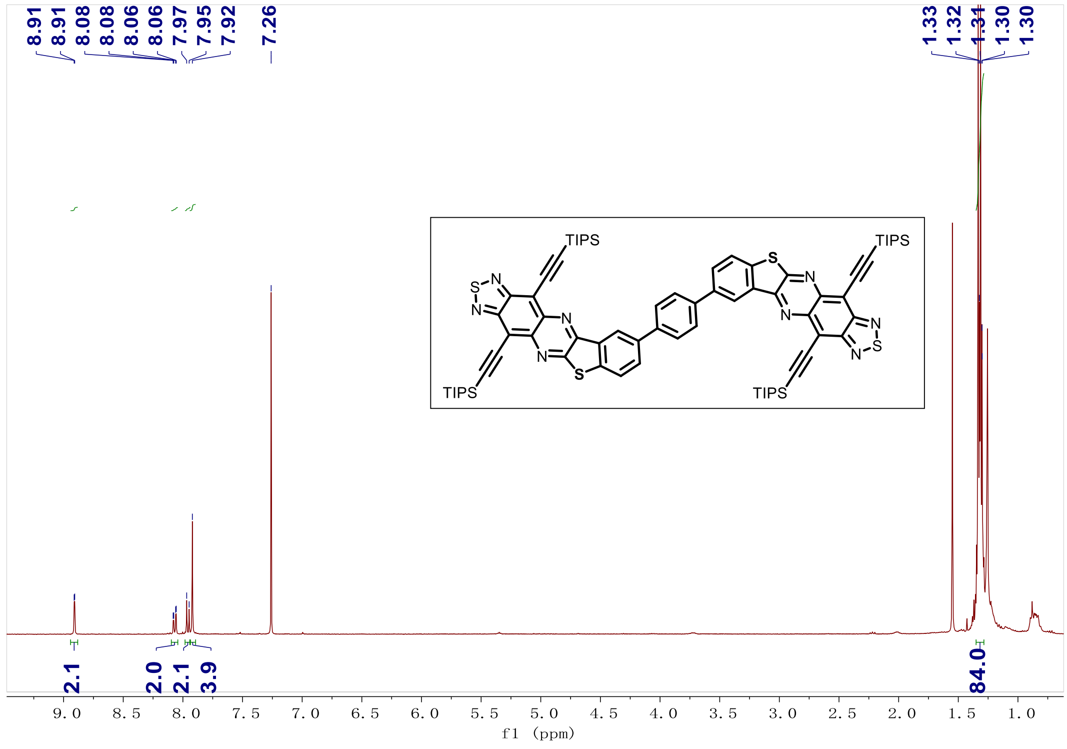

Supplementary Figure 5. ^{1}H-NMR of BTTQD 1.

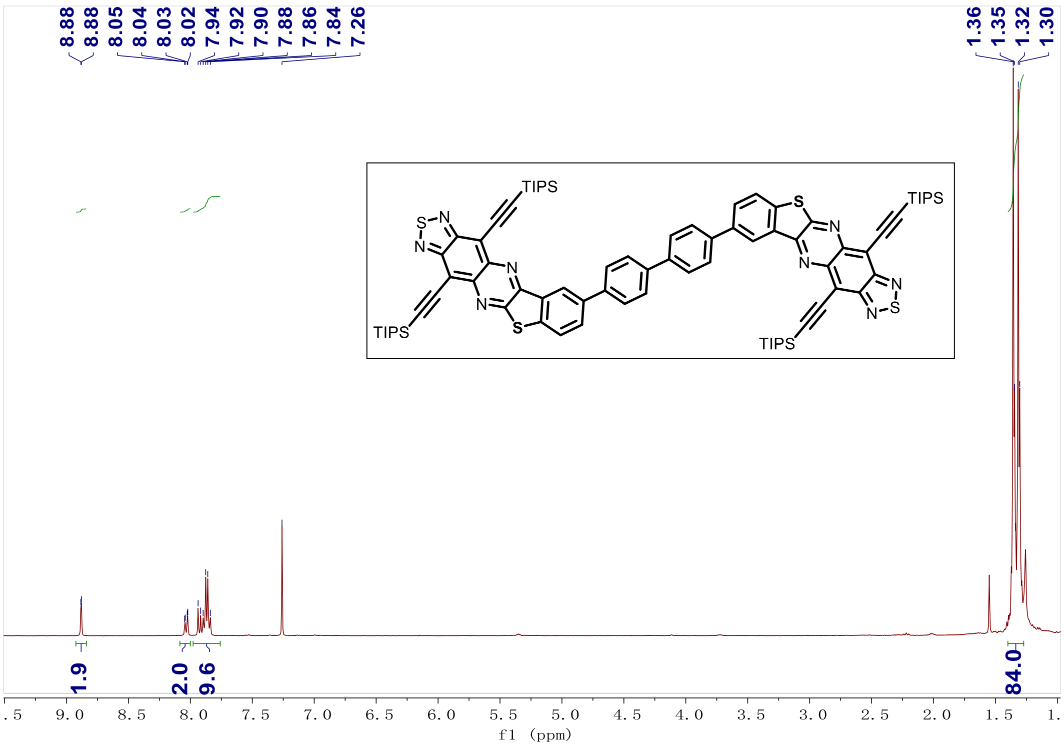

Supplementary Figure 6. ^{1}H-NMR of BTTQD 2.

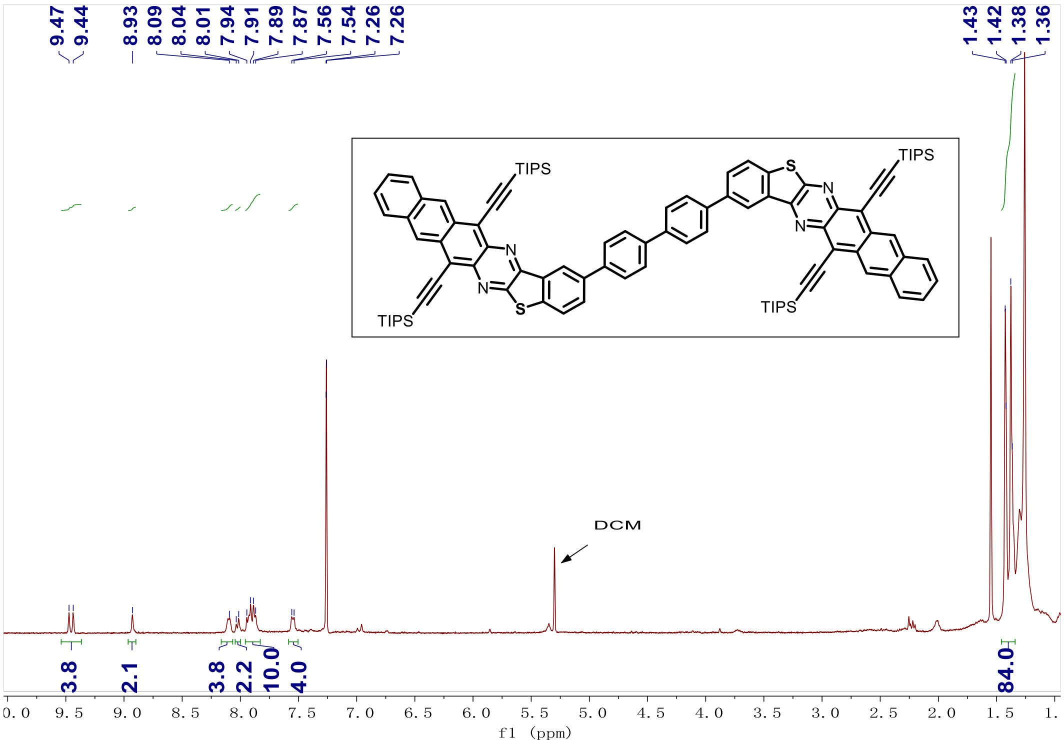

Supplementary Figure 7. ^{1}H-NMR of BTTQD 3.

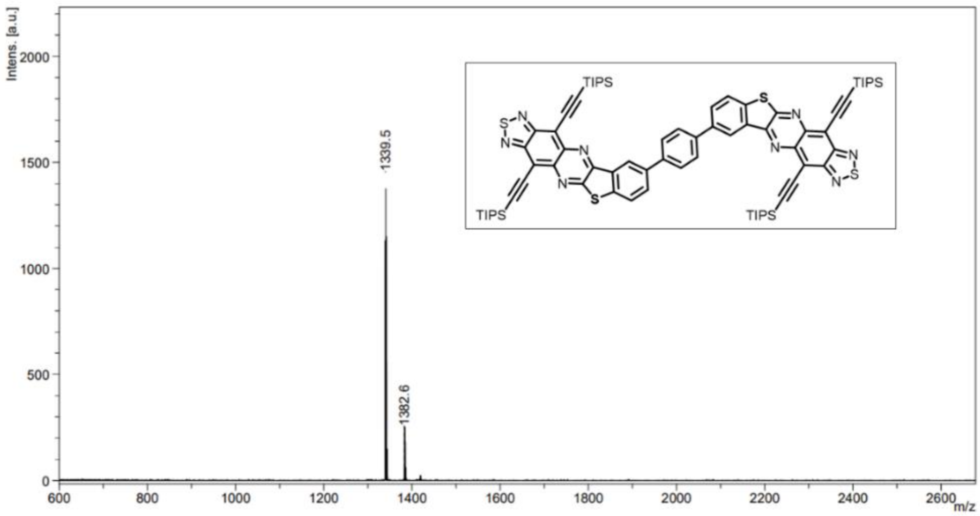

Supplementary Figure 8. Matrix assisted laser desorption ionization time of flight mass spectrometry (MALDI-TOF-MS) of BTTQD 1.

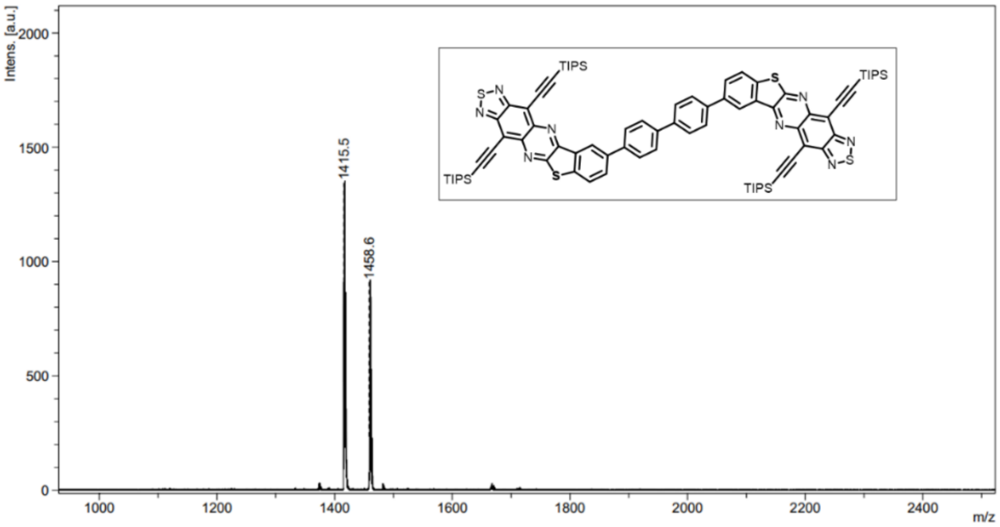

Supplementary Figure 9. Matrix assisted laser desorption ionization time of flight mass spectrometry (MALDI-TOF-MS) of BTTQD 2.

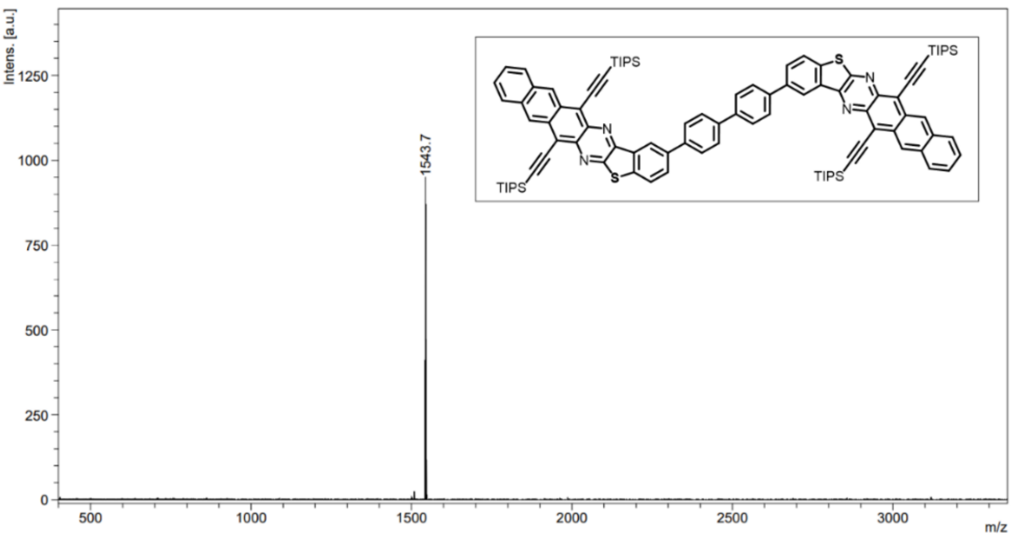

Supplementary Figure 10. Matrix assisted laser desorption ionization time of flight mass spectrometry (MALDI-TOF-MS) of BTTQD 3.

1.8 Synthesis of F127-NH_2

The amino-terminated F127 was synthesized following a procedure reported previously with some modifications.[5-6] Briefly, 5 g of F127 was dissolved in 100 ml of anhydrous dichloromethane under ice water bath. Next, 1.8 mL Et_3N was added to the reaction system. Then, 1 ml of methoxy chloride was added dropwise to the reaction solution, which was stirred in N_2 atmosphere for 24 hours. After removing the solvents by rotary evaporation, the crude products were redissolved by 5 mL DCM and reprecipitated by 200 mL diethyl ether. Subsequently, ammonia water was added to the product and stir vigorously for 3 days. Lastly, F127-NH_2 was extracted with DCM, concentrated by rotary evaporation, and lyophilized. The products were confirmed by ninhydrin colorimetry.

1.9 Preparation of TTA nanomicells with and without Cy 7

TTA nanomicells without Cy7: First, hydrophobic IR806 and BTTQD 2 molecules encapsulated into an amphiphilic polymer F127-NH_2 via a conventional film hydration method.[7-8] Briefly, IR806, BTTQD 2 and F127-NH_2 were dissolved in chloroform as stock solutions at a concentration of 1×10^{-4} M for IR806 and BTTQD 2 and 60 mg/mL for F127-NH_2. Subsequently, stock solutions of 100 μL IR806, 1mL

BTTQD annihilators, and 12 mL F127-NH_2 were mixed, followed by sonication for 5 min. Followed by adding an additional chloroform of 10 mL, the mixture was dried under vacuum in a rotary evaporator at 37 °C to form a lipidic film. After the organic solvents were removed completely, 5 mL PBS (PH = 7.4) was added into the obtained lipidic film and was sonicated for 0.5 h to obtain a clear aqueous solution of IR806/BTTQD 2 nanomicells.

TTA nanomicells with Cy7: Cy7 dyes were covalently linked to the surface of IR808/BTTQD **2** nanomicells by an amide reaction according to the reported literature with some modifications.[2, 9] Briefly, Cy7 dyes (10 mg) were dissolved in 10 mL EtOH containing EDC (96 mg, 0.03 mmol) and NHS (86 mg, 0.75 mmol). After stirring for 30 minutes, 2 mL aqueous solutions of IR808/BTTQD 2 nanomicells (10 mg/mL) were added into the EtOH solutions and stirred overnight. The products were collected by centrifugation followed by several times washing to remove excess reactant. The as-prepared TTA-NMs were dried and re-dissolved in PBS to form clear solutions, and the solutions will be used directly for biology experiments without further modification.

1.10 Preparation of various ROS/RNS stock solutions

$ONOO^-$: $ONOO^-$ stock solution was prepared by mixing the precursors of H_2O_2 (0.7 M, 1.5 ml), HCl (0.6 M, 1.5 ml), $NaNO_2$ (0.6 M, 3 ml) and NaOH (1.5 M, 3 ml), and then put into the refrigerator at –20 °C for standby. The concentration of $ONOO^-$ was calculated according to Beer-Lambert Law.

ClO^-: Dilute the NaClO solution (4-5%) directly with deionized water and put it into the refrigerator at –20 °C for standby. The concentration of hypochlorite was calculated according to its absorbance at 209 nm ($\varepsilon = 350\ M^{-1}cm^{-1}$).

Superoxide anion: Superoxide anion ($O_2\bullet^-$) was prepared according to the reaction between xanthine and xanthine oxidase mixture at room temperature. The concentration of $O_2\bullet^-$ was calculated using its absorbance at 256 nm ($\varepsilon = 2686\ M^{-1}cm^{-1}$).

H_2O_2: 30% H_2O_2 was diluted with deionized water to produce the desired concentrations.

•OH: 1 mM •OH was prepared by Fenton reaction between $FeCl_2$ (2 mM in water) and H_2O_2 (1 mM).

2. Supplementary Discussion

2.1 Kinetic analysis of transient absorption spectra[10-13]

The rate constants of intersystem crossing (k_{ISC}) were calculated according to equation (2):

$$k_{ISC} = \frac{1}{\tau_{rise}} \ (2)$$

where τ_{rise} values were obtained by fitting the kinetic curves in Figure 3a and 3b.

The rate constants of triplet-to-triplet energy transfer (k_{TTET}) were calculated according to equation (3):

$$k_{TTET} = \frac{1}{\tau_{T_1,D\text{-}A}} - \frac{1}{\tau_{T_1,D}} \ (3)$$

where $\tau_{T_{1}IR806}$ values were obtained from fitting of the kinetic curves in Figure 3c and 2d.

The efficiency of triplet-to-triplet energy transfer (Φ_{TTET}) were calculated according to equation (4):

$$\Phi_{TTET} = 1 - \frac{\tau_{T1,\ D\text{-}A}}{\tau_{T1,\ D}} \ (4)$$

where $\tau_{T1,\ D\text{-}A}$ is the lifetime of donor (IR806) triplet in presence of acceptor (BTTQD), and $\tau_{T1,\ D}$ is the lifetime of donor triplet in absence of acceptor. The corresponding values obtained from the kinetic curves of TA spectra.

The rate constants oxygen quenching of T_1 were calculated according to equation (4):

$$k_{[oxygen]} = \frac{1}{\tau_{aerated}} - \frac{1}{\tau_{deaerated}} \ (5)$$

where $\tau_{aerated}$ and $\tau_{deaerated}$ values were obtained from the triplet state lifetimes of IR806-BTTQD 2 sample under aerated and deaerated conditions.

For Φ_{TTA}, it is described by the relation:

$$I_{UC}\ (t) \propto \left(\frac{1\text{-}\Phi_{TTA}}{e^{k_T t}\text{-}\Phi_{TTA}}\right)^2 \ (6)$$

where k_T is the decay rate of BTTQD 2 triplet and I_{UC} is the intensity of delayed fluorescence at a specific time. The fit of the experimental data yields $k_T = 2.34 \times 10^4$ s^{-1} and 2.43×10^4 s^{-1}, and Φ_{TTA} = 95.3% and 95.9% in the absence and presence of

oxygen, respectively.

2.2 Kinetic analysis from classical steady/transient -state measurements

The quenching efficiency of the emitter molecule to the sensitizer triplet emission (phosphorescence) can be quantitatively explained by the Stern−Volmer (SV) equation:[14]

$$\frac{I_0}{I} = k_{SV}[M]+1= k_{TTET}\times\tau_0[M]+1 \quad (7)$$

$$\frac{1}{\tau} = k_{TTET}[M]+ \frac{1}{\tau_0} \quad (8)$$

where I_0/τ_0 and I/τ are the phosphorescence intensities/lifetimes of IR806 in the absence and presence of BTTQD emitter, [M] is the molar concentration of analytes BTTQD emitter, k_{SV} and k_{TTET} are the Stern-Volmer constant and triplet-to-triplet energy transfer rate constant, respectively. It is important to note that the rate constant derived from the Stern-Volmer equation is concentration-dependent, so for comparison with transient absorption results, a uniform concentration should be introduced for the final rate calculation. Supplementary Figure 21 and 22 shows the Stern–Volmer plot of IR806 generated from the change in phosphorescence intensities and lifetimes as various amounts of BTTQD 2 were added to a fixed amount of IR806 in the absence and presence of oxygen. The steady-state phosphorescence emission intensities of a series of samples were recorded at 808 nm excitation, with the IR806 concentration fixed at 1×10^{-5} M, and the BTTQD 2 concentration varied from 0.1 to 1.0×10^{-4} M. The k_{SV} in deaerated and aerated conditions was calculated as 25900 M^{-1} and 15700 M^{-1}, respectively. Together with the triplet lifetime of the single molecule IR806 (τ_0 = 4.57 μs for deaerated sample and 3.01 μs for aerated sample), the k_{TTET} rate constant can be evaluated to be 5.66×10^9 M^{-1} s^{-1} and 5.22×10^9 M^{-1} s^{-1} in the absence and presence of oxygen, respectively. Thus, k_{TTET} can be evaluated as 5.66×10^5 s^{-1} and 5.22×10^5 s^{-1} in the absence and presence of oxygen when the employed emitter concentration is consistent with the transient absorption measurements (1.0×10^{-4} M). Similarly, based on the changes in the triplet lifetimes of IR806, k_{TTET} can also be calculated according to Eq. 8, whose values are 5.74×10^5 s^{-1} and 5.21×10^5 s^{-1} in the absence and presence of oxygen, respectively. Therefore, the TTET rate evaluated by

classical steady/transient-state measurements have a good correlation with the transient absorption results ($k_{TTET} = 5.56 \times 10^5\ s^{-1}$ and $5.15 \times 10^5\ s^{-1}$ in the absence and presence of oxygen).

2.3 Reliability assessment of experimentally observed TTET rates

In a previous paper, Monguzzi et al. established a kinetic model of upconversion emission and donor phosphorescent emission as functions of the photophysical parameters of the employed molecules [15-16] In order to verify the reliability of our study, we give the expression of the ratio of energy transfer rate under aerated ($\bar{k}_{TTET}$) and deaerated (k_{TTET}) conditions by following the same model. Specifically, when the excitation power density is below the TTA threshold, the main deactivation channel of the emitter triplet is self-quenching, and the ratio between $\bar{k}_{TTET}$ and k_{TTET} can be given by the following expression:

$$\frac{\bar{k}_{TTET}}{\mathrm{k}_{TTET}} = \left[\frac{k_{rad}^{D}}{\bar{k}_{rad}^{D}}\left(\frac{\bar{k}_{T}^{A}}{k_{T}^{A}}\right)^2 \frac{k_{exp}^{D}}{\bar{k}_{exp}^{D}} \frac{\bar{I}_A / \bar{I}_D}{I_A / I_D}\right]^{\frac{1}{2}} \quad (9)$$

where k_{rad}^{D} is the radiative decay rate of the donor (IR806), k_{exp}^{D} represents the triplet decay rate of the donor measured experimentally (equal to the sum of k_{T}^{D} and k_{TTET}), k_{T}^{A} is the triplet decay rate of the acceptor (BTTQD 2), I_A and I_D are the TTA-UC fluorescence of the acceptor and the phosphorescence of the donor, respectively. The bar (—) overhead the parameter indicates the parameter in the presence of oxygen. $\frac{k_{exp}^{D}}{\bar{k}_{exp}^{D}}$ can be given by the ratio of the time-resolved photoluminescence spectra intensities of the deaerated and aerated samples when the delay time $\tau = 0$. The lifetime of the acceptor triplet state (the inverse of k_{T}^{A}) can be estimated from the long component lifetime in the TTA-UC decay profile ($\tau_{T1}^{A} = 2\tau_{UC}$). Finally, I_A and I_D can be simply obtained from time-integrated photoluminescence intensities of steady state measurements. The corresponding parameters have been summarized in Supplementary Table 1.

Supplementary Table 1. The involved parameters obtained by classical steady/transient state measurements.

	k_{rad}^{D}	k_{exp}^{D}	k_{T}^{A}	$\frac{\bar{I}_A/\bar{I}_D}{I_A/I_D}$
deaerated	$2.06 \times 10^5\ s^{-1}$	$7.90 \times 10^5\ s^{-1}$	$2.34 \times 10^4\ s^{-1}$	1.31
aerated	$3.12 \times 10^5\ s^{-1}$	$8.33 \times 10^5\ s^{-1}$	$2.43 \times 10^4\ s^{-1}$	

According to Equation 9 and the parameters in Supplementary Table 1, $\frac{\bar{k}_{TTET}}{k_{TTET}}$ was estimated to be 0.941, in good agreement with the one of 0.926 estimated from TA measurement. These results indicate oxygen does not enhance TTET rate in our biocomponent TTA-UC system, different from the reported drastic enhancement of TTET rate in the presence of oxygen, by A. Monguzzi et al,[15] in typically investigated bicomponent TTA system (Donor: PtOEP, Acceptor: DPA). One of the most likely reasons is that the triple state decay rate of the BTTQD emitter used in this work was almost not influenced by surrounding oxygen molecules, in sharp contrast to that of the DPA emitter in which the $T_1 \rightarrow S_0$ transition dipole moment was drastically enhanced by oxygen molecules.[15] This is reasonable as DPA emitter molecule containing polycondensated aromatic rings has a near-completely forbidden $T_1 \rightarrow S_0$ transition (decay rate, 200 s^{-1}), while the BTTQD 2 emitter in this work, containing a dimer structure with heteroatoms inserted into the framework of polycyclic aromatic hydrocarbons, is relaxed in spin forbiddance (decay rate, 23400 s^{-1}), therefore having negligible influence by the paramagnetic oxygen molecules.

2.4 Oxygen quenching efficiency for the triplets of sensitizer and annihilator

The oxygen quenching efficiency η_{O2} for the donor triplet can be estimated:

$$\eta_{O2} = k_{O_2} \Big/ (k_{O_2} + k_{TTET} + k_{T}^{D}) \quad (10)$$

in which $k_{TTET} = 5.56 \times 10^5\ s^{-1}$ and $k_{T}^{D} = 2.19 \times 10^5\ s^{-1}$. As a result, the oxygen quenching efficiency of the donor triplet was calculated to be η_{O2}=12.7%.

Likewise, the oxygen quenching efficiency of the acceptor triplet can be evaluated using:

$$\eta_{O2} = {k_{O_2}} \Big/ {(k_{O_2} + k_{TTA} + k_T^A)} \quad (11)$$

in which $k_T^A = 2.34 \times 10^4\ s^{-1}$ and k_{TTA} is dependent on the excitation light irradiance. At an extreme case where $k_{TTA} = 0$ such as TTA process at extreme low light irradiance where the first order process is significantly higher than the second order process, the maximum triplet quenching efficiency was evaluated to be $\eta_{O2} = 3.7\%$, clearly indicating that there is nearly none oxygen quenching for the acceptor triplets.

2.5 Measurement of upconverstion quantum yield (UCQY)

The upconversion quantum yield (Φ_{UC}) was calculated according to equation[1, 17]:

$$\Phi_{UC} = \Phi_{std}\left(\frac{A_{std}}{A}\right)\left(\frac{I}{I_{std}}\right)\left(\frac{N}{N_{std}}\right)^2 \quad (12)$$

where A_{std} and A are the numbers of photons absorbed by the standard reference sample (with known quantum yield) and the measured sample, respectively; *I* and I_{std} represent the integrated luminescence intensities of the measured sample and the standard reference sample, respectively; *N* and N_{std} are the average refractive index of the solvent used for dissolving the measured sample and the referenced standard sample, respectively. Here, indocynine green (ICG) dye in DMSO, with a known quantum yield of 12%, was utilized a standard reference to quantify Φ_{UC}.

For TTA-UC, the upconversion quantum yield Φ_{UC} can be determined using equation 13 with a theoretical maximum of 50%.[18]

$$\Phi_{UC} = 0.5 f \Phi_{ISC} \Phi_{TTET} \Phi_{TTA} \Phi_{F} \quad (13)$$

where Φ_{ISC}, Φ_{TTET}, Φ_{TTA}, Φ_{F} are the quantum efficiencies of the intersystem crossing of the sensitizer, triplet-to-triplet energy transfer between the sensitizer and annihilator, triplet-triplet annihilation (TTA) process of the annihilator, and singlet fluorescence of the annihilator, respectively. The f represents the spin-statistical factor, which illustrates

the probability of two triplets annihilating to form a usable singlet. For most cases with $E_{T_2} > 2E_{T_1}$, the spin-statistical factor could be assumed to be $f = 2/5$. We determined Φ_{ISC} = 72.2%, Φ_{TTET} = 60.8%, Φ_F = 27.0%, Φ_{TTA} = 95.3%. Therefore, Φ_{UC} could be theoretically estimated to be ~2.3%, very close to the experimentally determined one of 2%.

2.6 Photoluminescence quantum yield (PLQY) of IR806 and BTTQD 1-3

The effect of oxygen on fluorescence quantum yield of sensitizer and emitter molecules was investigated. The absolute quantum yield was calculated using the following expression:

$$\Phi = \frac{\int L_{emission}}{\int E_{reference} - \int E_{sample}} \qquad (14)$$

where $L_{emission}$ is the emission spectrum of the sample, E_{sample} is the spectrum of the incidence excitation light not absorbed by the sample, and $E_{reference}$ is the spectrum of the excitation light not absorbed by the reference in the sphere. Pure organic solvent (chloroform) was used as reference samples and xenon lamps are used as excitation light sources. The aerated sample was prepared in an atmospheric environment and the deaerated sample was prepared in a glove box using an anhydrous solvent. The fluorescence quantum yield values of IR806 and BTTQD 1-3 are summarized in Supplementary Table 2 and 4.

Supplementary Table 2. Key parameters of the compounds in the absence and presence of oxygen.

	deaerated	aerated
$\tau_{S_1(S)}$	1.18 ns[a]	1.15 ns[a]
$\tau_{S_1(A)}$	18.4 ns[a]	18.4 ns[a]
$\tau_{T_1(S)}$	4.57 μs[b]	3.01 μs[b]
$\tau_{T_1(A)}$	42.8 μs[c]	41.2 μs[c]
$QY_{S_1(S)}$	7.0%[d]	6.8%[d]
$QY_{S_1(A)}$	62.8%[d]	62.7%[d]
$QY_{T_1(S)}$	44.0%[e]	–
$QY_{T_1(A)}$	–	–
$\Phi_{UC,\ g}$	2.3%[f]	2%[f]
Φ_{ISC}	44.0%[g]	–
Φ_{TTET}	71.8%[h]	60.8%[h]
Φ_{TTA}	95.3%[i]	95.9%[i]

[a] The data are derived from classical transient state measurements in chloroform c = 1 * 10^{-5} M^{-1}, λ_{ex} = 785 and 405 nm for sensitizer and emitter, respectively. [b] The data are determined with femtosecond/nanosecond transient absorption spectroscopy in chloroform. [c] The triplet lifetime of the emitter is estimated by the formula $2\tau_{UC} = \tau_T^A$. [d] Fluorescence quantum yields were measured at room temperature using an integrating sphere. [e] The triplet quantum yield at low temperatures is assumed to be equal to the ISC efficiency (vide infra). [f] Upconversion quantum yield (normalized) with ICG as standard (12% in DMSO). [g] It is evaluated by the decay kinetics of the singlet state and the rise kinetics of the triplet state in femtosecond transient absorption spectra. [h] The data are determined with nanosecond transient absorption spectra in chloroform. [i] The TTA efficiency is estimated by the Eq. 6. − Not observed. The letter S stands for sensitizer, A for annihilator.

We are unable to directly measure the phosphorescence yield (Φ_{PL}) of IR806 triplet using an integrating sphere. Instead, when all non-radiative pathways are assumed to be suppressed at 77K, the triplet quantum yield of IR806 in the absence of oxygen should equal the intersystem crossing efficiency ($\Phi_{PL}= \Phi_{ISC}$) of 44.04% (see Figure 3a). With the introduction of oxygen, a new triplet decay channel is activated (Φ_{O_2}), and the evaluation of Φ_{PL} should consider oxygen quenching ($\Phi_{PL}= \Phi_{ISC}\big(1$-

Φ_{O_2})). According to the decay rate of IR806 triplet lifetime after the introduction of oxygen, the Φ_{O_2} can be estimated to be 34.14% (see Figure 3c in manuscript file). Therefore, the Φ_{PL} of IR806 under aerated condition could be assessed as 29.01%. For BTTQD emitters, the large energy gap between T_1 and S_1 and the almost completely spin-forbidden nature of the $T_1 \rightarrow S_0$ transition results in phosphorescence with very low quantum yield, theoretically close to ~0, regardless of the presence of oxygen molecules.[15]

3. Supplementary Figures

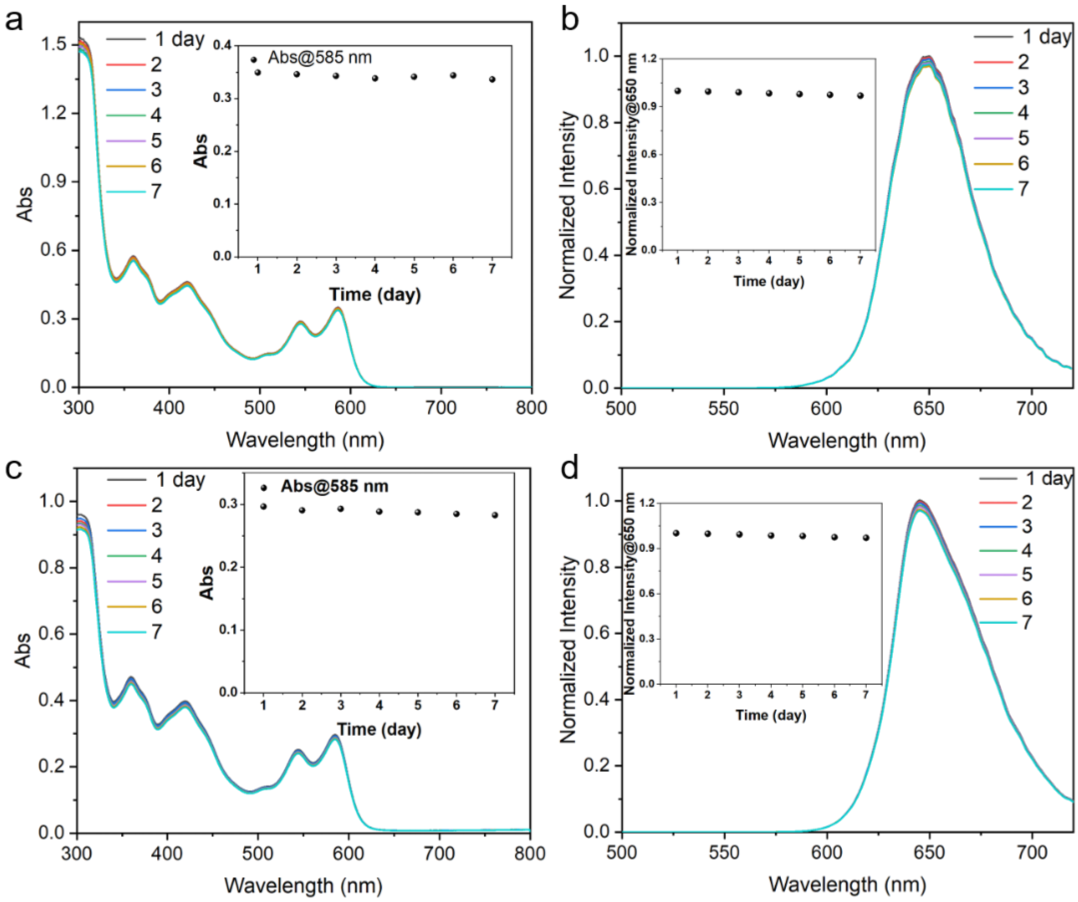

Supplementary Figure 11. The stability of BTTQD 2 stored for different durations in aerated and deaerated environments. Absorption (**a, c**) and emission (**b, d**) spectra of BTTQD 2 in deaerated (**a, b**) and aerated (**c, d**) chloroform observed over 7 days (c = 1×10^{-5} M).

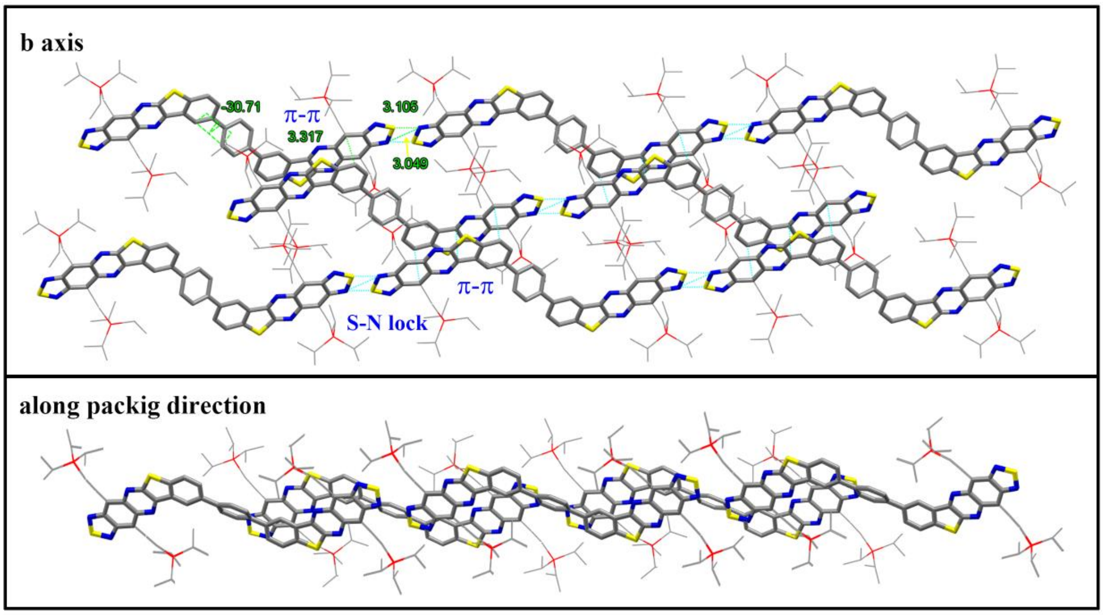

Supplementary Figure 12. Analyzed single crystal structure of BTTQD 1.

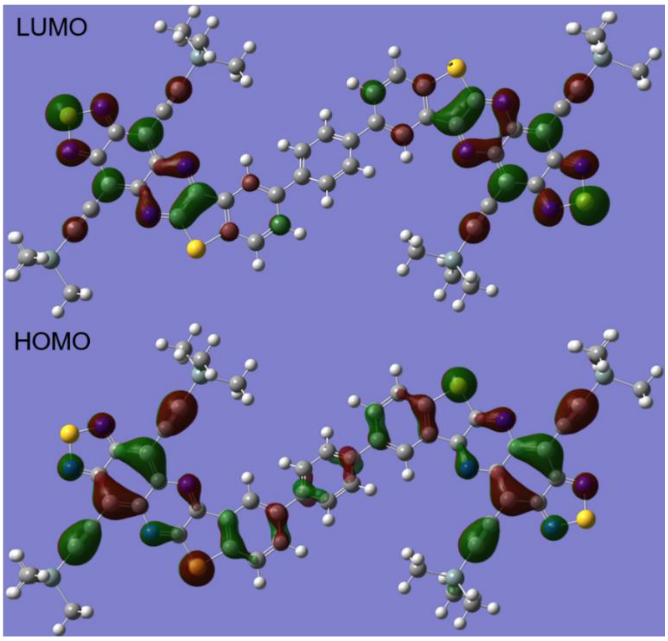

Supplementary Figure 13. Electron density maps of the LUMO and HOMO molecular orbitals of BTTQD 1.

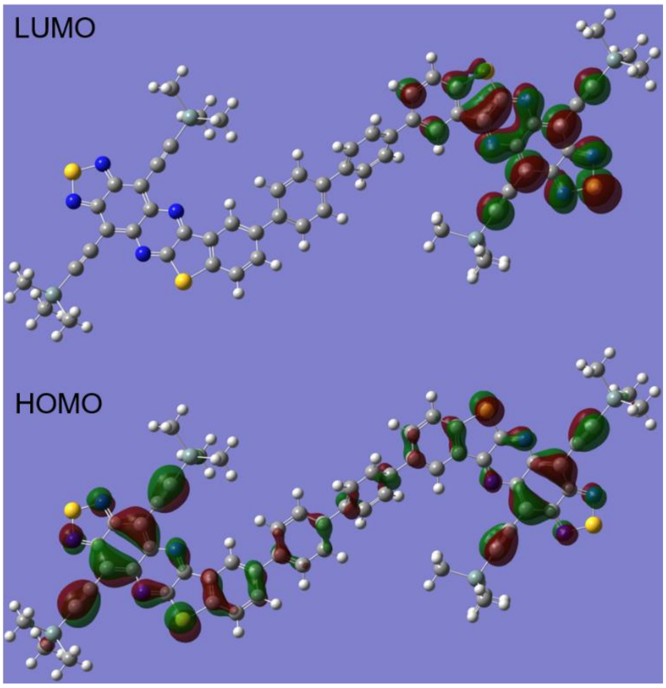

Supplementary Figure 14. Electron density maps of the LUMO and HOMO molecular orbitals of BTTQD 2.

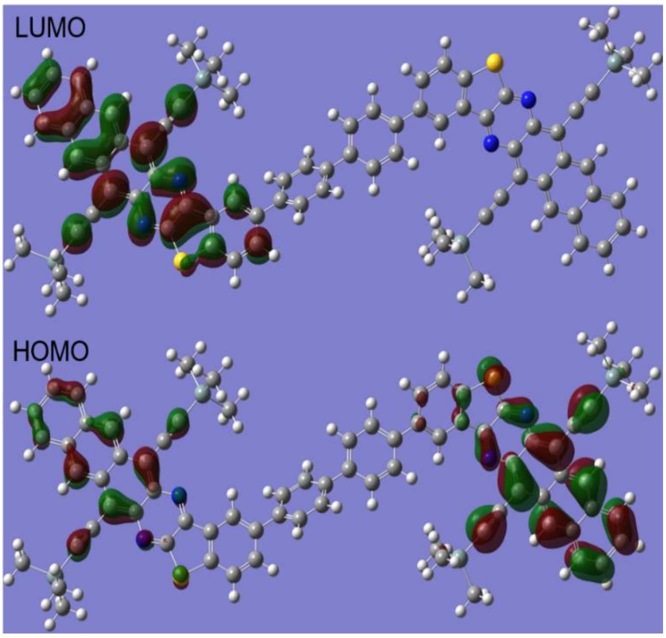

Supplementary Figure 15. Electron density maps of the LUMO and HOMO molecular orbitals of BTTQD 3.

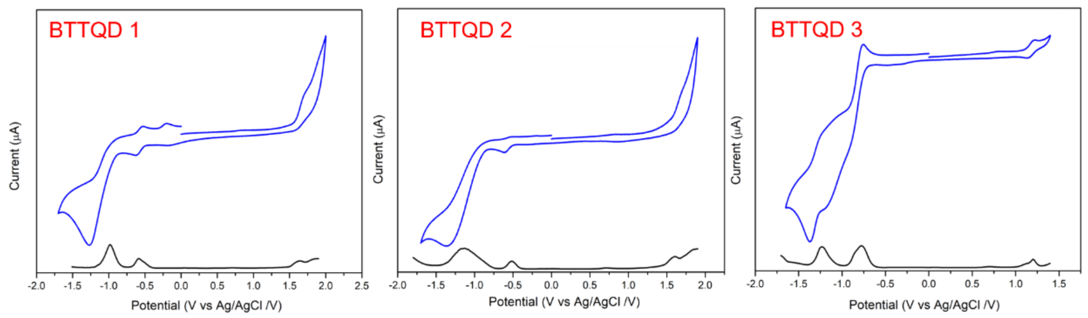

Supplementary Figure 16. Cyclic voltammogram (CV) and differential pulse voltammetry (DPV) curves of BTTQD 1-3 from left to right, respectively. The blue closed curve is the cyclic voltammetry curves, while the black is the DPV curves. There is no significant difference in the oxidation potential and reduction potential between BTTQD 1 and BTTQD 2, however, BTTQD 3 has poorer redox reversibility, which may be the reason why BTTQD 3 is less stable than BTTQD 1 and 2.

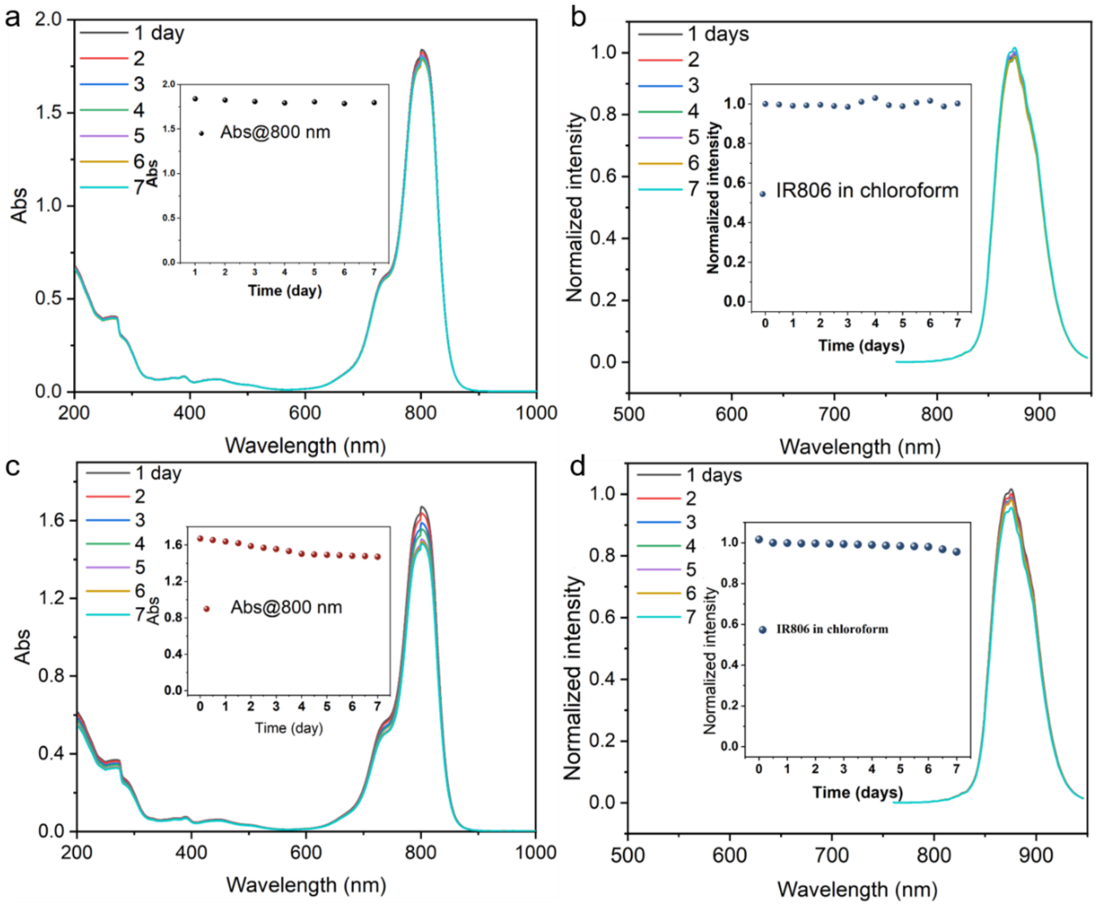

Supplementary Figure 17. The stability of IR806 stored for different durations in aerated and deaerated environments. Absorption (**a, c**) and emission (**b, d**) spectra of IR806 in deaerated (**a, b**) and aerated (**c, d**) chloroform over 7 days ($c = 1 \times 10^{-5}$ M).

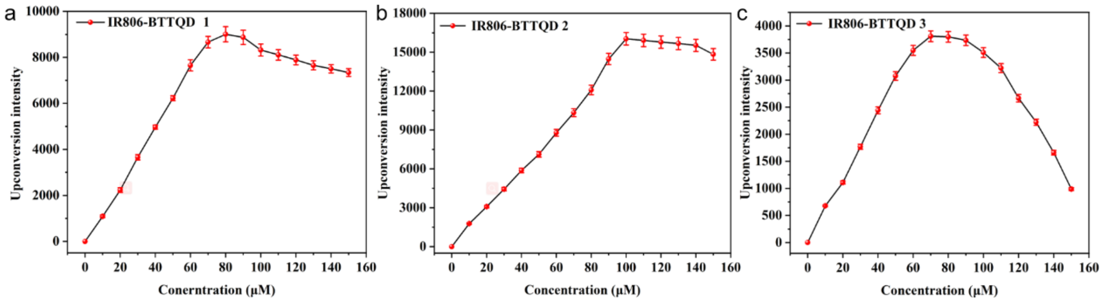

Supplementary Figure 18. Determination of the optimal concentrations of sensitizer and annihilator. Measured upconversion luminescence with a fixed amount of IR806 (1×10^{-5} M) and varied concentrations of BTTQD 1 (**a**), BTTQD 2 (**b**), and BTTQD 3 (**c**). The optimal concentrations were determined to be 80, 100, and 70 μM for BTTQD 1, 2, and 3 in aerated chloroform, respectively. The upconversion intensity is presented as mean ± standard deviations (SD) (n = 3).

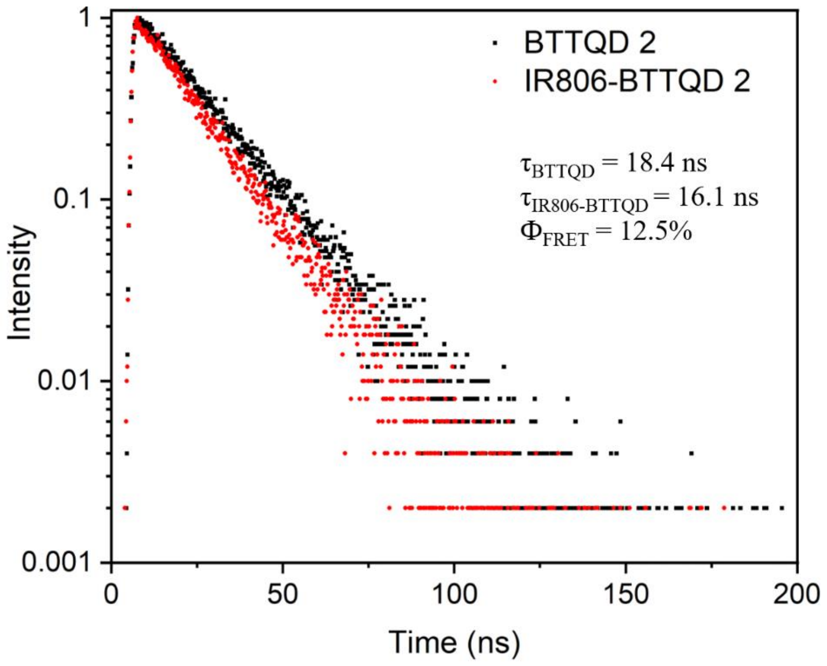

Supplementary Figure 19. Determination of the reverse energy transfer efficiency from BTTQD 2 to IR806. Decay lifetimes of the spin-singlet state of BTTQD 2 in the freely-diffusing form (black, 18.4 ns) and in the IR806-BTTQD 2 (red, 16.1 ns) mixture (chloroform, $c_{IR806} = 1 \times 10^{-5}$ M, $c_{BTTQD\ 2} = 1 \times 10^{-4}$ M).

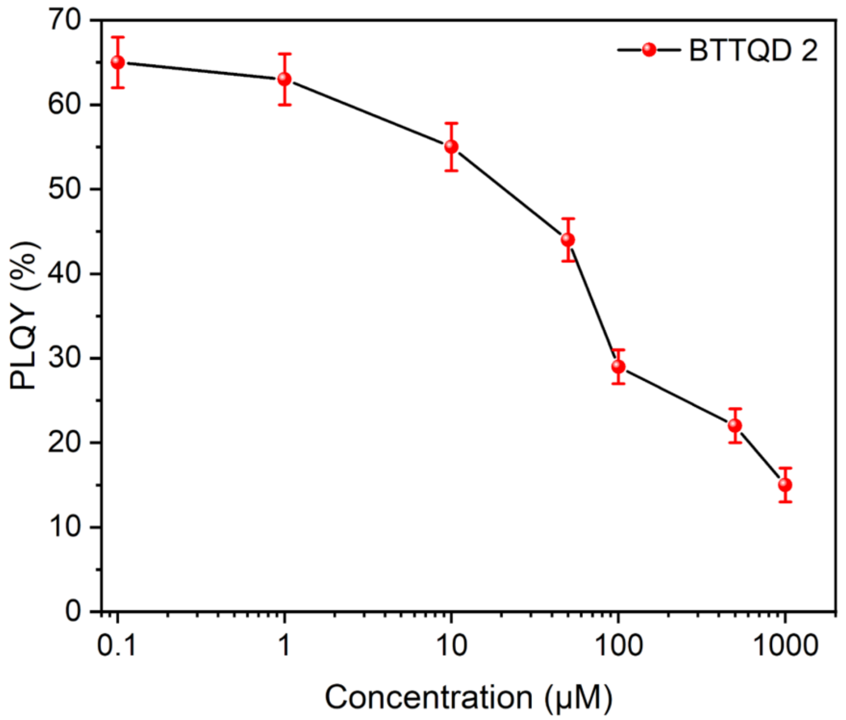

Supplementary Figure 20. PLQY of BTTQD 2 annihilator as a function of its concentration in chloroform. PLQY is presented as mean ± standard deviations (SD) (n = 3).

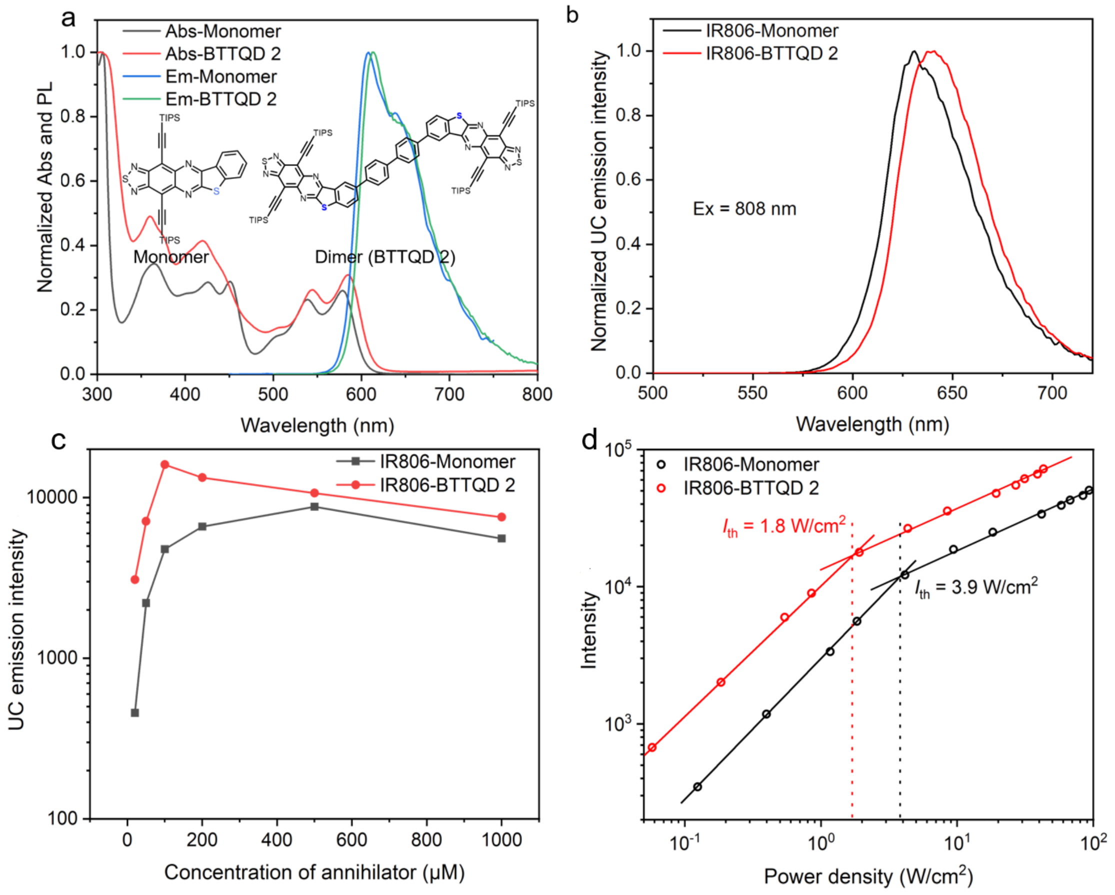

Supplementary Figure 21. Comparison of the spectral properties of monomeric and dimeric BTTQD 2 and their upconversion emission properties in TTA. (**a**) Normalized absorption and photoluminescence (λ_{ex} = 450 nm) spectra of BTTQD 2 and its corresponding monomer. (**b**) Normalized upconverted emission spectra of solutions containing IR806 as sensitizer and monomer/BTTQD 2 as annihilators (IR806 : 10 μM, monomer or BTTQD 2 : 200 μM). (**c**) TTA-UC emission intensities as a function of the annihilator concentration with fixed IR806 concentration of 10 μM. (**d**) Logarithmic plots of upconversion emission intensities against excitation power densities for IR806-monomer and IR806-BTTQD 2 in chloroform, respectively (IR806: 10 μM, monomer or BTTQD 2 : 200 μM).

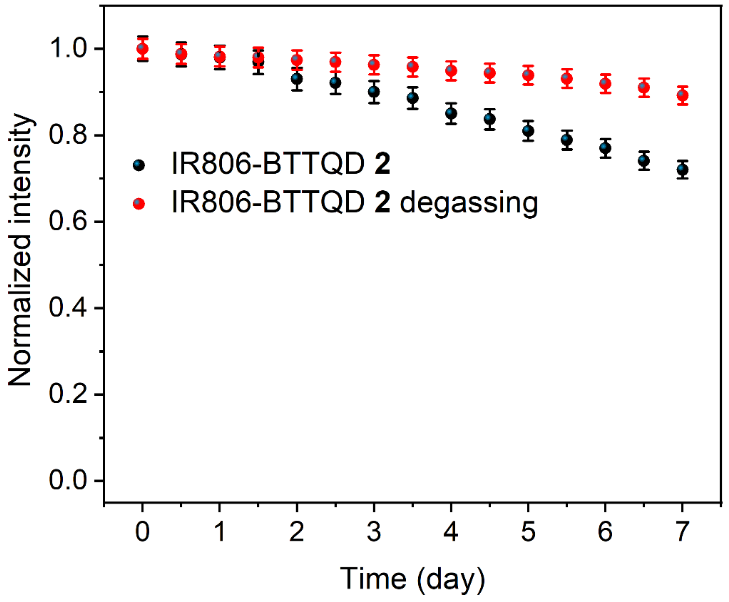

Supplementary Figure 22. Emission stabilities of upconversion in IR806-BTTQD 2 chloroform solution with/without degassing; $c_{IR806} = 1 \times 10^{-5}$ M, $c_{BTTQD\ 2} = 1 \times 10^{-4}$ M. The luminescence intensity is presented as mean ± standard deviations (SD) (n = 3).

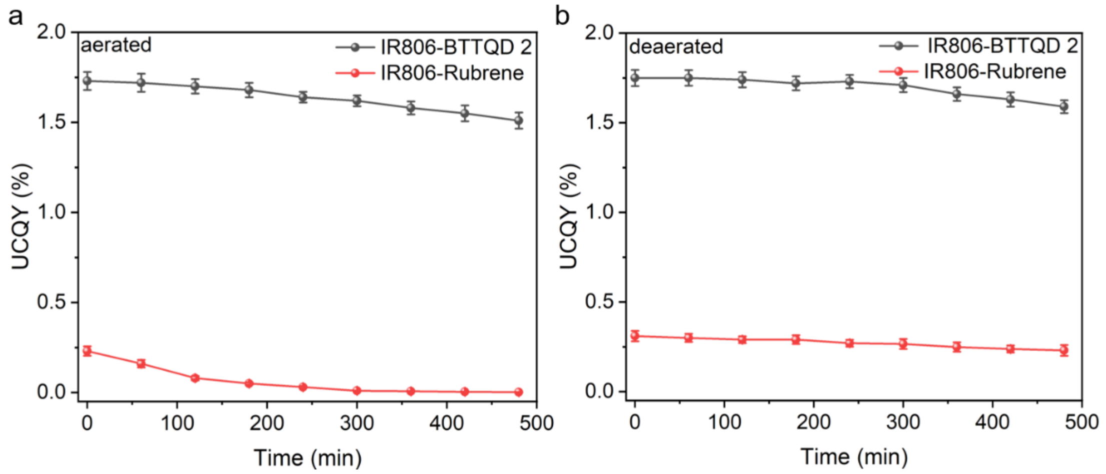

Supplementary Figure 23. The evolution of UCQYs in IR806-BTTQD 2 (in chloroform) and IR806-rubrene (in toluene) systems. Time-dependent UCQYs are presented in the presence (**a**) and absence (**b**) of oxygen under continuous laser irradiation at 808 nm (100 mW/cm^2). UCQYs were measured at power density of 10 W/cm^2 with $c_{\mathrm{IR806}} = 1 \times 10^{-5}$ M, $c_{\mathrm{Rubrene/BTTQD\ 2}} = 1 \times 10^{-4}$ M. UCQYs are presented as mean ± standard deviations (SD) (n = 3).

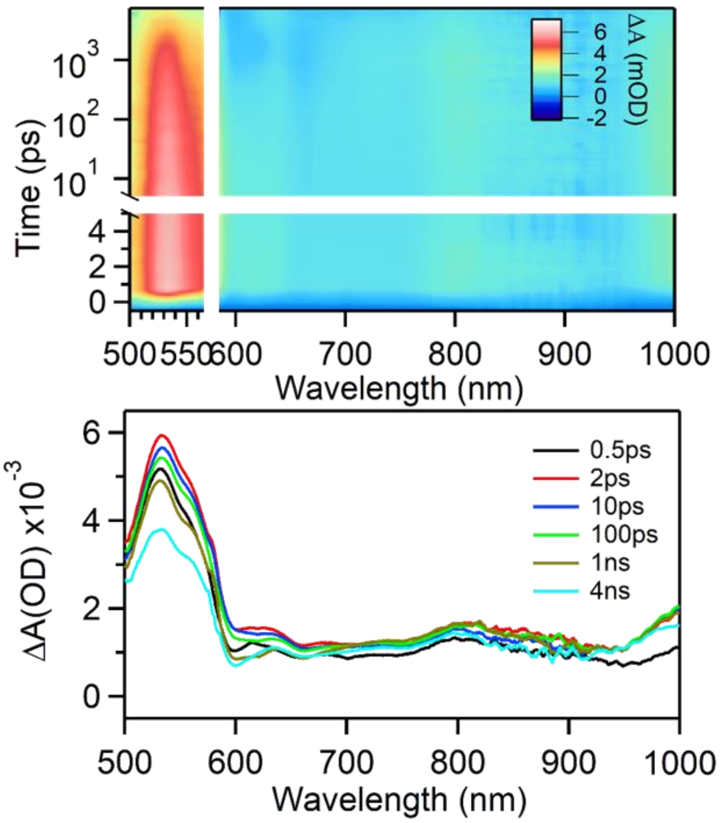

Supplementary Figure 24. Femtosecond transient absorption spectra of BTTQD 2 (1 × 10^{-4} M) in chloroform (λ_{ex}=580 nm) under degassing. The positive peak around 530 nm that rises first and then decays, belongs to the triple excited state absorption of BTTQD 2.

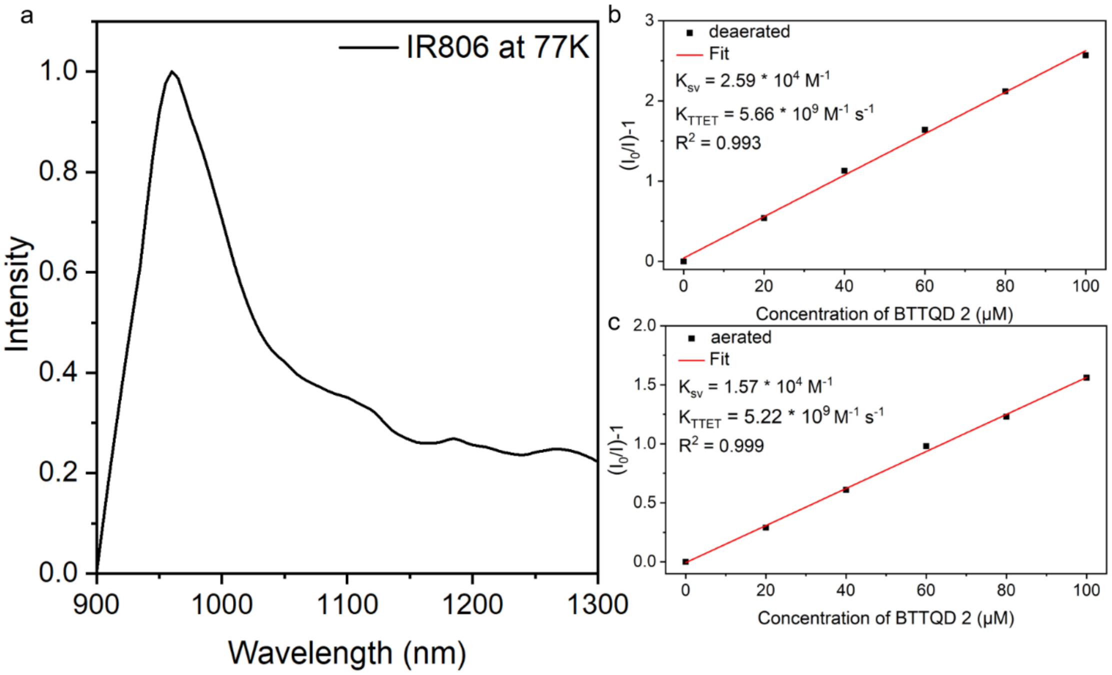

Supplementary Figure 25. Determination of TTET rate through classical steady-state measurements. (**a**) Measured phosphorescence spectra of IR806 (chloroform, 1×10^{-5} M). (**b, c**) Plotting the intensity of IR806 phosphorescence peaked at 970 nm against various concentrations of BTTQD 2 in the absence (**b**) and presence (**c**) of oxygen molecules. The solid red lines represent Stern-Volmer fitting. IR806 concentration was fixed at 1×10^{-5} M, while BTTQD 2 concentration was varied from 0.1 to 1.0×10^{-4} M. Phosphorescence spectra were measured at 77K under continuous-wave 808 nm laser excitation.

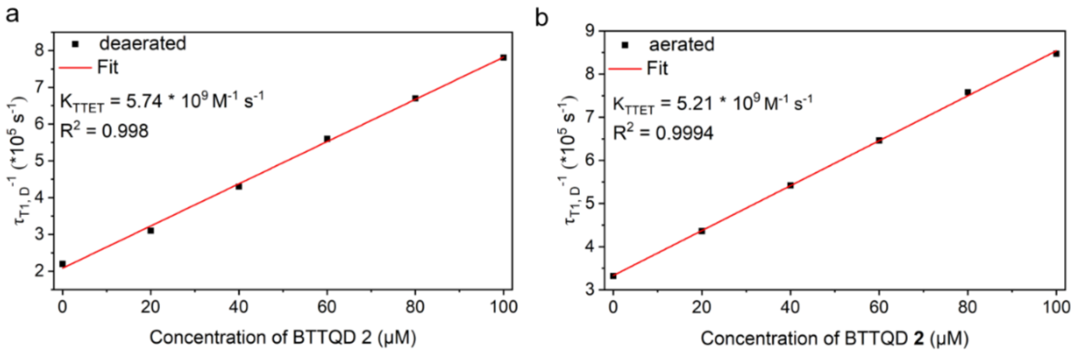

Supplementary Figure 26. Determination of TTET rate through Stern-Volmer relation. Plotting IR806 phosphorescence lifetimes at 970 nm against various concentrations of BTTQD 2 in the absence (**a**) and presence (**b**) of oxygen molecules. The solid lines represent Stern-Volmer fitting. IR806 concentration was fixed at 1×10^{-5} M, while BTTQD 2 concentration was varied from 0.1 to 1.0×10^{-4} M. The experiments were performed at 77 K under 808 nm laser excitation operating in pulsed mode.

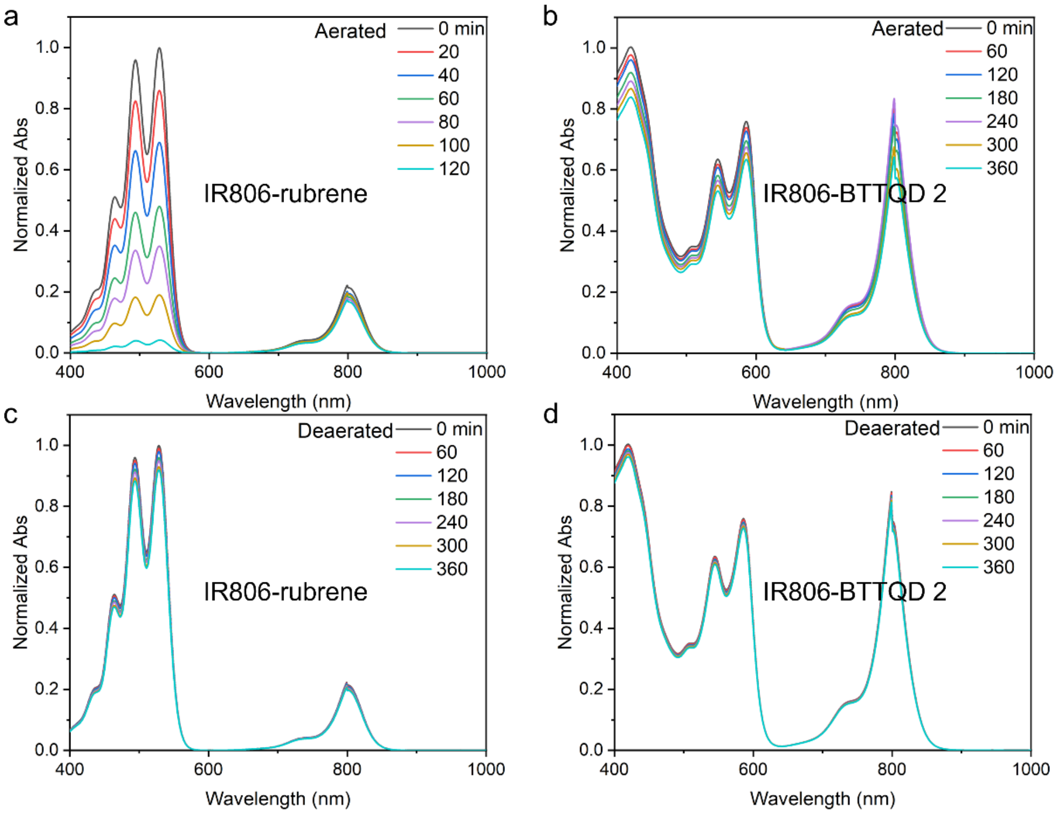

Supplementary Figure 27. Evaluating photodegrading processes of IR806-BTTQD 2 and IR 806-rubrene systems. Absorption spectra of (**a, c**) IR806-rubrene (in toluene) and (**b, d**) IR806-BTTQD 2 (in chloroform) in aerated (**a, b**) and deaerated (**c, d**) solution during long-time laser irradiation (λex = 808 nm, 100 mW/cm^2). $c_{\mathrm{IR806}} = 1 \times 10^{-5}$ M, $c_{\mathrm{Rubrene/BTTQD\ 2}} = 1 \times 10^{-4}$ M.

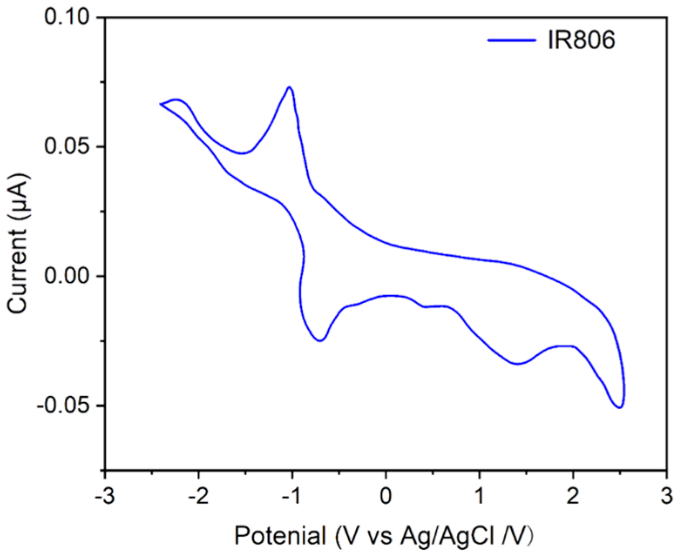

Supplementary Figure 28. Cyclic voltammogram (CV) curves of IR806.

For cyanine dyes represented by IR806, the conjugated chain part of its structure is most vulnerable to singlet oxygen attack. Kanofsky and Sima et al. have demonstrated that the resistance of cyanine dyes to singlet oxygen depends to a certain extent on the position of dye oxidation potential.[19-20] We performed a cyclic voltammetry experiment on IR806. As shown in Supplementary Figure 24, the reduction potential (ΔG_{red}) value was measured as −0.41 eV for IR806. We then indirectly evaluated oxidation potential by looking at the highest occupied molecular orbital (HOMO) and (lowest unoccupied molecular orbital) LUMO levels of IR806:[21-22]

$$E_{LUMO} = -e(\Delta G_{red} + 4.4) \quad (14)$$

$$E_{HOMO} = -e(\Delta G_{ox} + 4.4) \quad (15)$$

$$E_{HOMO} = E_{LUMO} - E_g \quad (16)$$

E_g was calculated to be 1.31eV from the position of the absorption boundary of the IR806 absorption spectrum. Therefore, the oxidation potential of IR806 was evaluated to be 0.9 V. On the other hand, the oxidation potential of BTTQD 2 was evaluated to be 1.4 V. Both values are significantly higher than that of rubrene (0.4 V)[23] and DPA (0.2 V)[24] emitter, thereby showing high stability against oxygen damage than rubrene emitter. This can be clearly seen through distinct solution color changes over time in aerated solutions (Figures 2b and 2d).

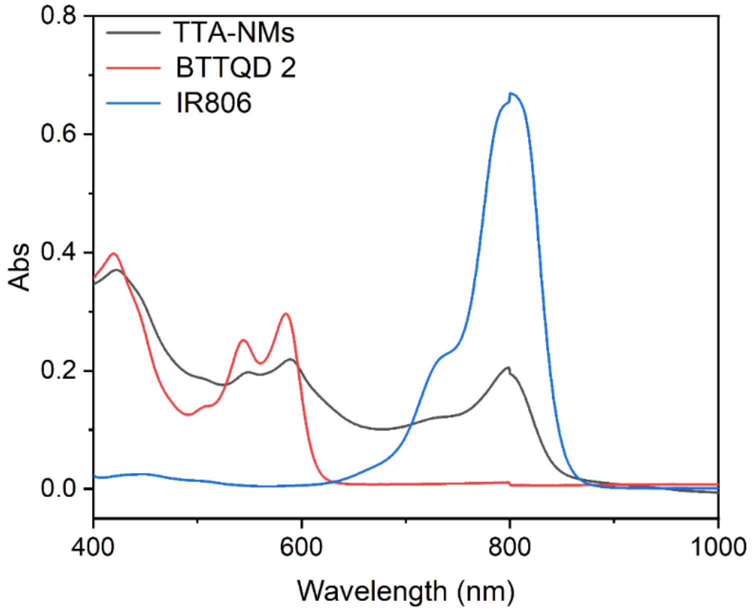

Supplementary Figure 29. Absorption spectra of IR806 (in toluene), BTTQD 2 (in chloroform), and TTA-NMs (in PBS).

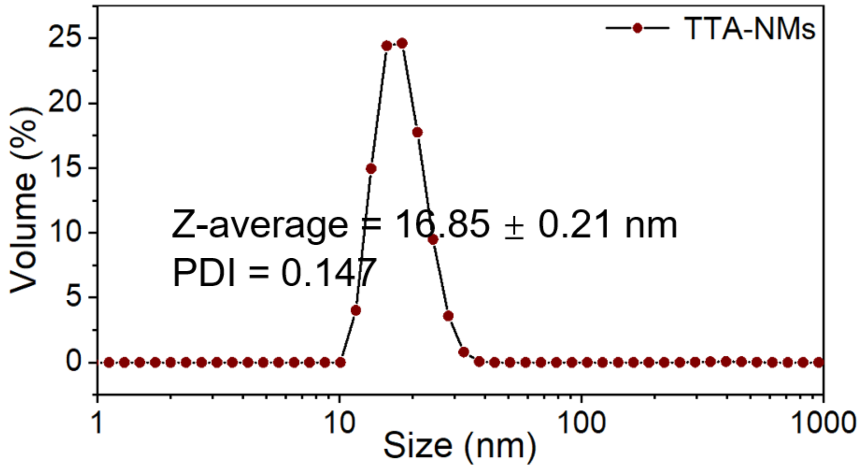

Supplementary Figure 30. Dynamic light scattering (DLS) analysis of TTA-NMs.

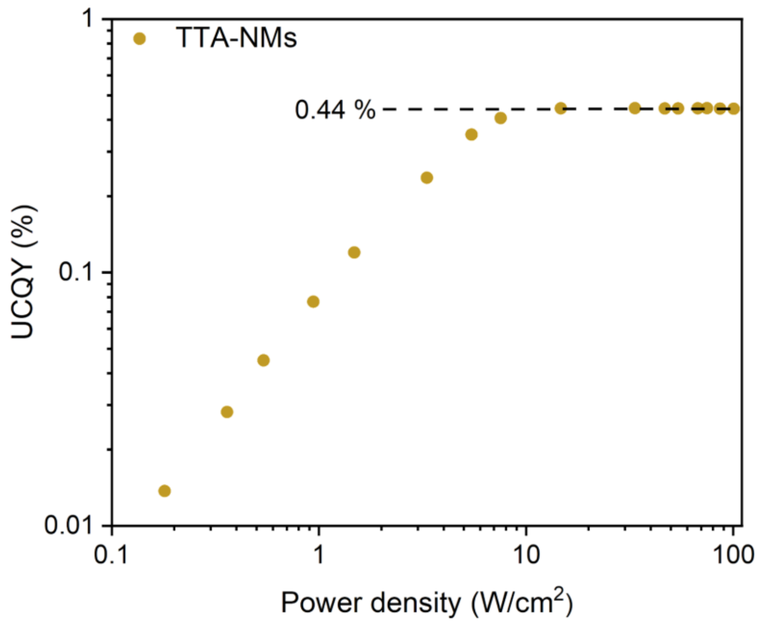

Supplementary Figure 31. UCQY of TTA-NMs in PBS as a function of the incident power density under 808 nm excitation.

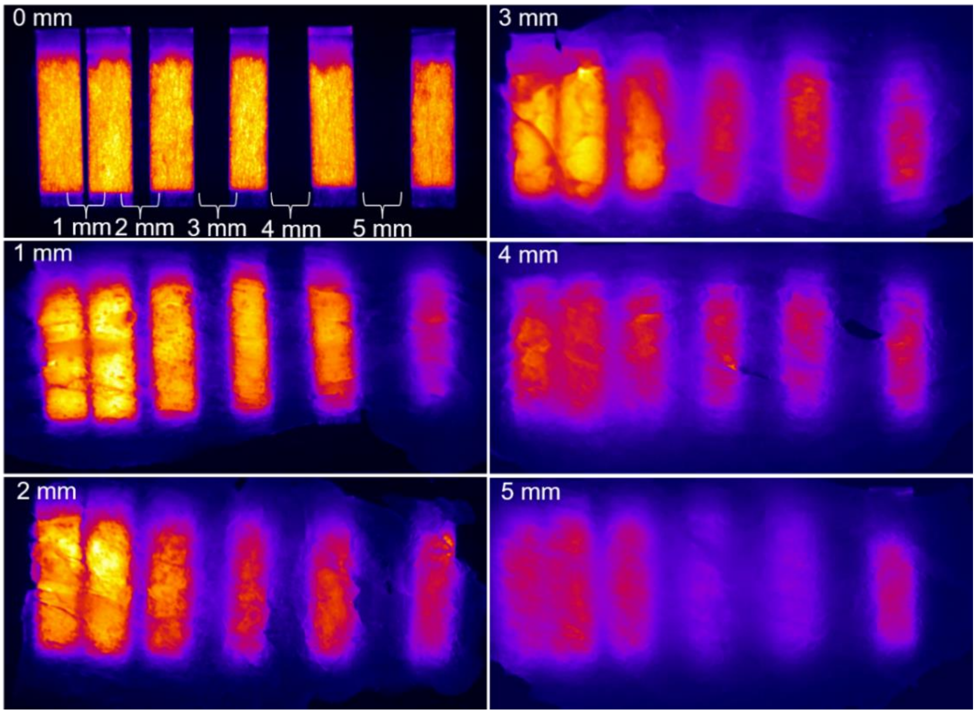

Supplementary Figure 32. Determination of lateral upconversion imaging resolution through varying thickness of pork tissue (0, 1, 2, 3, 4 and 5 mm). These filter papers were soaked with TTA-NMs and placed with defined distances (1, 2, 3, 4 and 5 mm) for TTA-UC imaging. λ_{ex} = 808 nm, 100 mW/cm^2, exposure time: 50 ms.

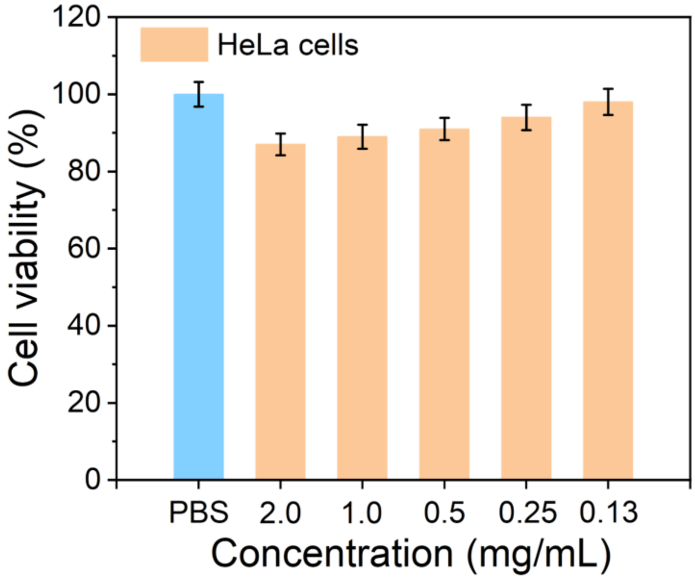

Supplementary Figure 33. Cell viability (%) of Hela cells treated with TTA-NMs at different concentrations (0-2 mg/mL), The data is represented as mean ± standard deviations (SD) (n = 3).

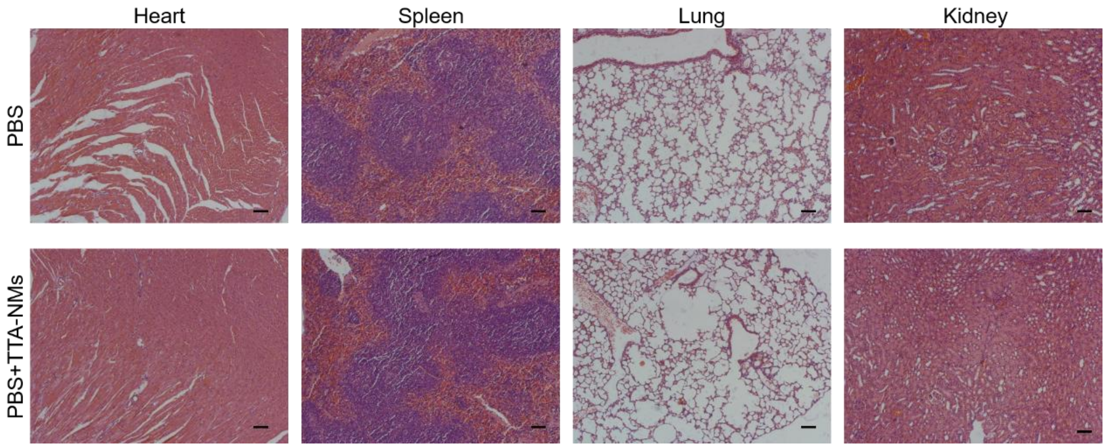

Supplementary Figure 34. H&E-stained histological sections of the harvested organs (heart, spleen, lung and kidney) from healthy mice and the mice treated with TTA-NMs, scale bar: 50 µm. Independent experiments were repeated three times, yielding similar results.

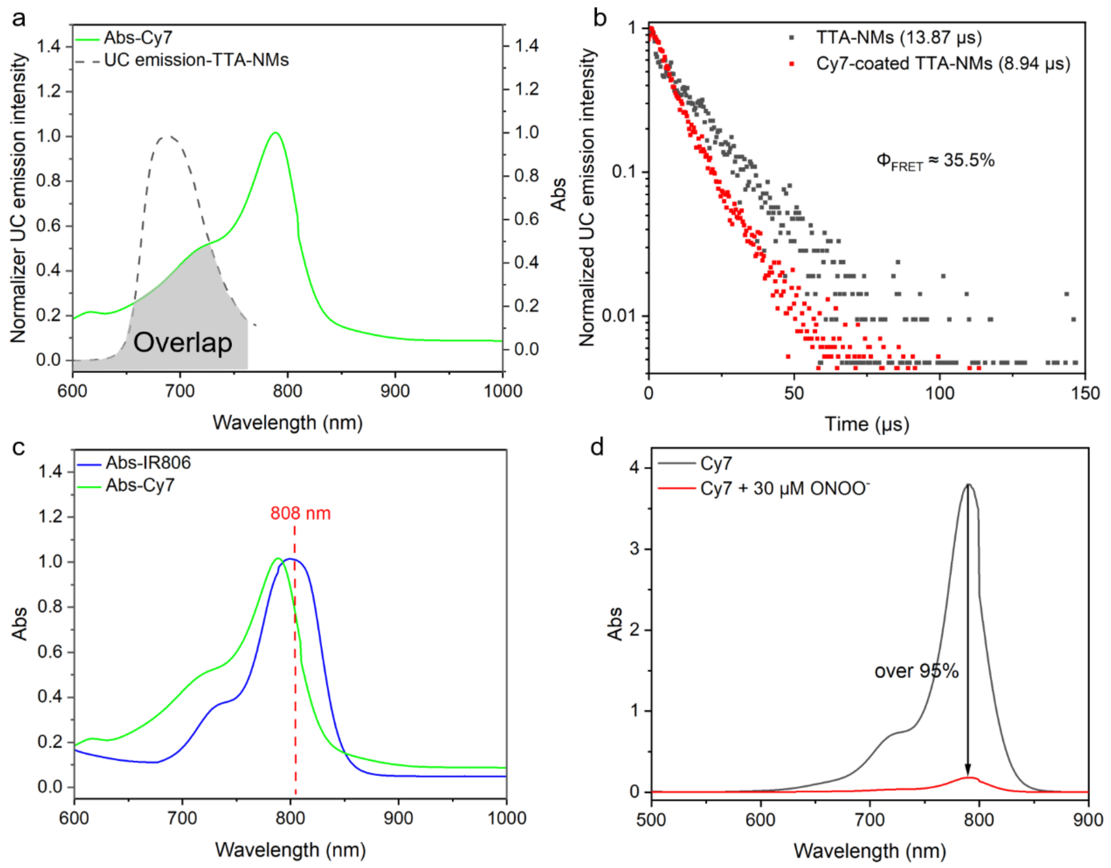

Supplementary Figure 35. Mechanistic study of Cy7 quenching in TTA-NMs upconversion emission. (**a**) Absorption spectrum of Cy7 (1×10^{-5} M) and upconversion emission spectrum of TTA-NMs (5 mg/mL) under photoexcitation at 808 nm (100 mW/cm^2). (**b**) UC emission lifetimes of TTA-NMs in PBS (5 mg/mL) before and after the introduction of Cy7. (**c**) Absorption spectra of Cy7 and IR806 in H_2O, the red dotted line represents the location of 808nm excitation wavelength. (**c**) Changes of Cy7 absorption spectra before and after 30 μM $ONOO^-$ treatment. Upon introducing Cy7 to the surface of TTA-NMs, the lifetime decreased from 13.87 to 8.94 μs, indicating the occurrence of a Förster resonance energy transfer (FRET) process with an efficiency of approximately 35.5%. However, this efficiency alone is insufficient to explain the entirety of the TTA-UC quenching effect (> 90%) in TTA-NMs (Figure 4d). The competitive absorption effect of the excitation light between Cy7 and IR 806 should contribute to about 64.5% of the observed quenching process.

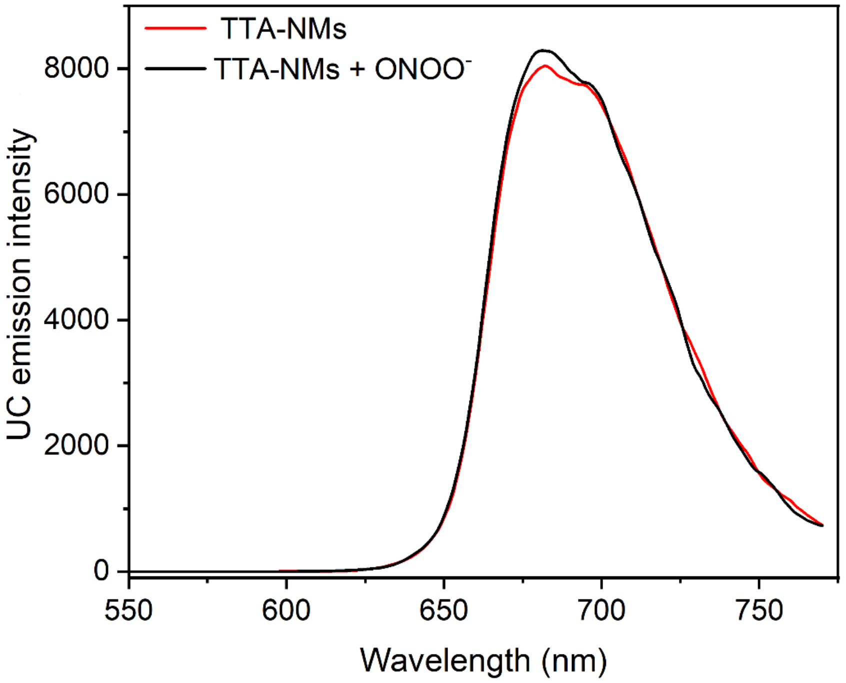

Supplementary Figure 36. The change of upconversion emission intensities of TTA-NMs (5 mg/mL, without Cy7 coating) toward $ONOO^-$ (30 μM). The upconversion emission spectra were measured after 1 minute when the probe was treated by $ONOO^-$.

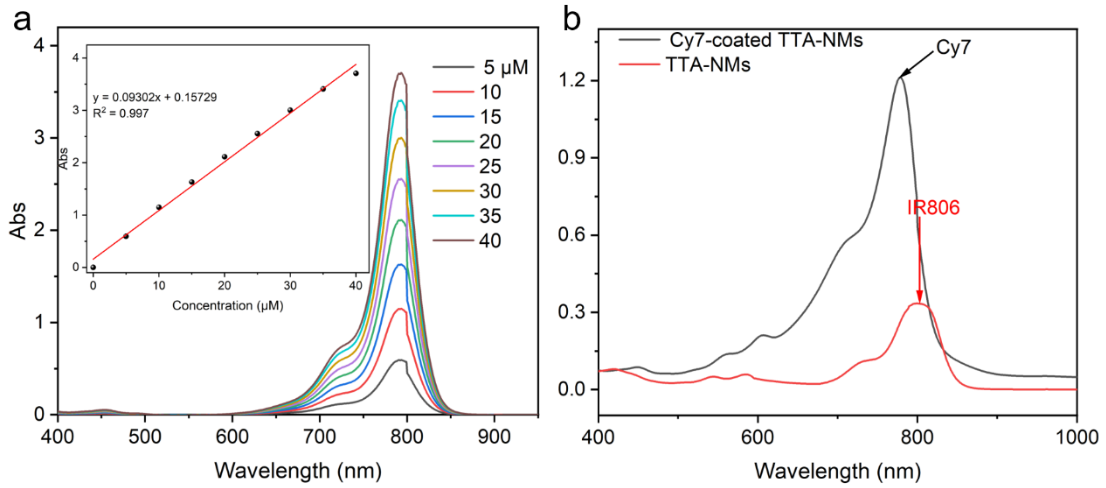

Supplementary Figure 37. Evaluation of the loading efficiency of Cy7 on the surface of TTA-NMs based on quantitative absorption method. (**a**) The absorption spectra of Cy7 with different concentrations (in chloroform). Inset: the standard curve for the absorbance of Cy7 at 790 nm. (**b**) The absorbance change of TTA-NMs before and after loading Cy7.

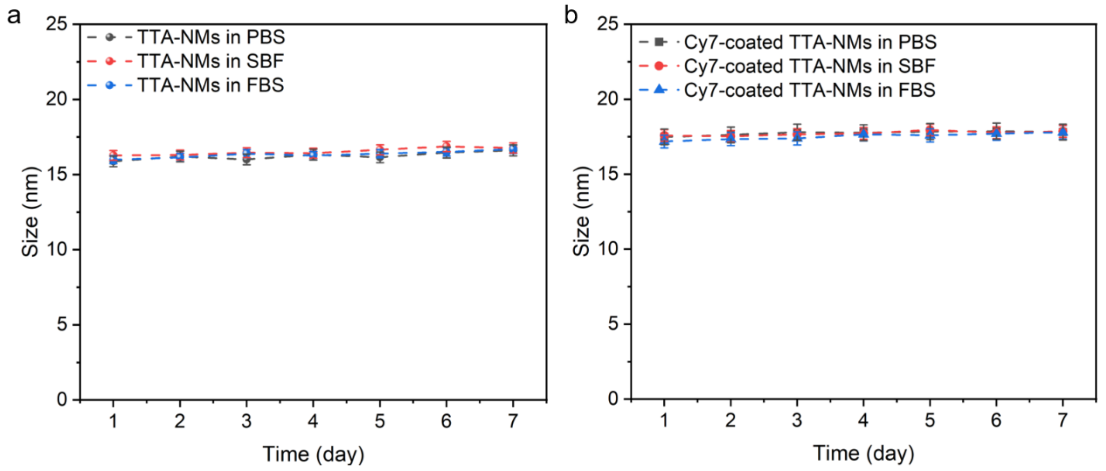

Supplementary Figure 38. Colloidal stability evaluation of TTA-NMs in saline. The particle size distributions of TTA-NMs (**a**) and Cy7-coated TTA-NMs (**b**) over a period of 7 days in PBS buffer (PH = 7.4), simulated body fluid (SBF, PH = 7.4) and fetal bovine serum (FBS, 10%). The size results are presented as mean ± standard deviations (SD) (n = 3).

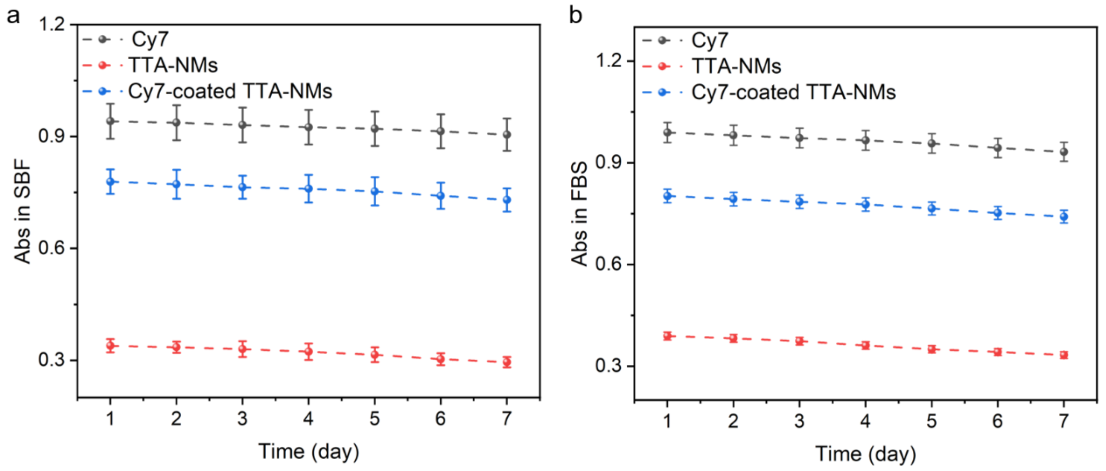

Supplementary Figure 39. Colloidal stability evaluation of TTA-NMs in saline through absorbance measurement of solutions. Time dependence of absorption from Cy7 dye, TTA-NMs and Cy7-coated TTA-NMs over a period of 7 days in SBF (**a**, PH = 7.4) and FBS (**b**, 10%). The absorption results are presented as mean ± standard deviations (SD) (n = 3).

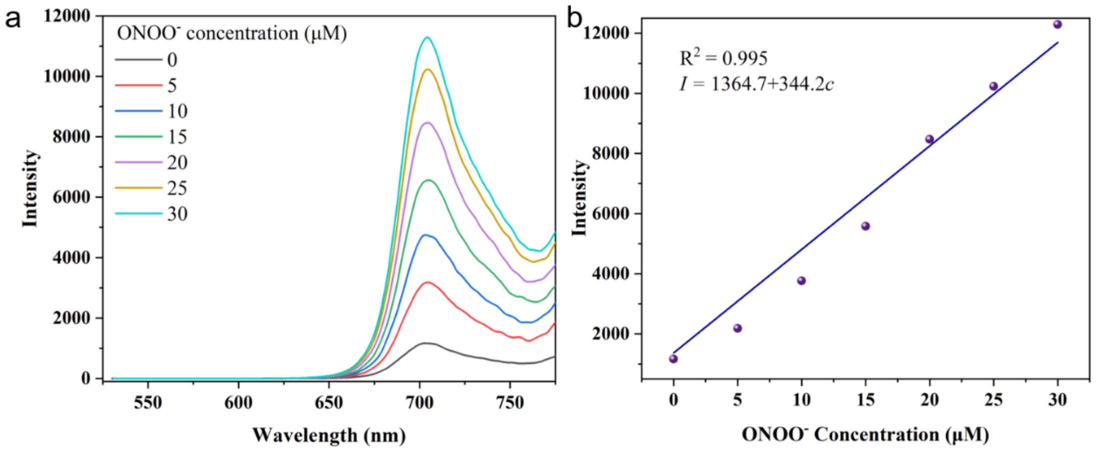

Supplementary Figure 40. Quantitative analysis experiment of $ONOO^-$ induced recovery of upconversion fluorescence on TTA-NMs. (**a**) Measured $ONOO^-$ concentration dependent upconversion emission spectra of Cy7-coated TTA-NMs aqueous solutions (0.2 mg/mL). (**b**) The plot of upconversion emission intensities at 700 nm with various $ONOO^-$ concentrations (0~30 μM).

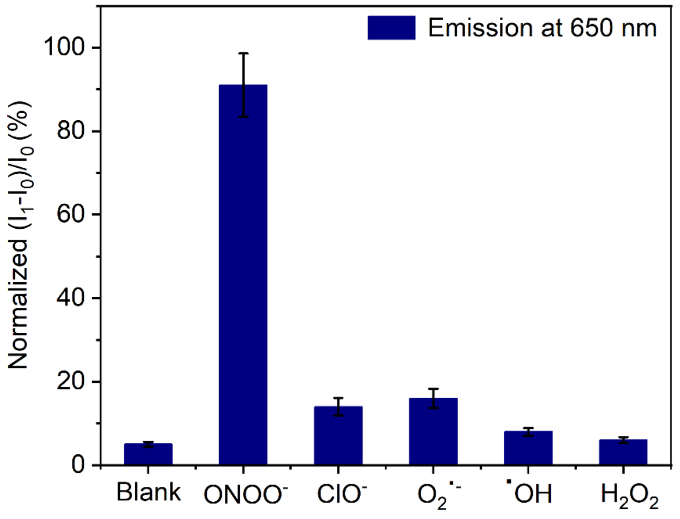

Supplementary Figure 41. The change of upconversion emission intensities of Cy7-coated TTA-NMs (5 mg/mL in PBS) toward various analytes (20 μM). The upconversion emission spectra were acquired 1 minute after treating Cy7-coated TTA-NMs with different ROS/RNS. The results are presented as mean ± standard deviations (SD) (n = 3).

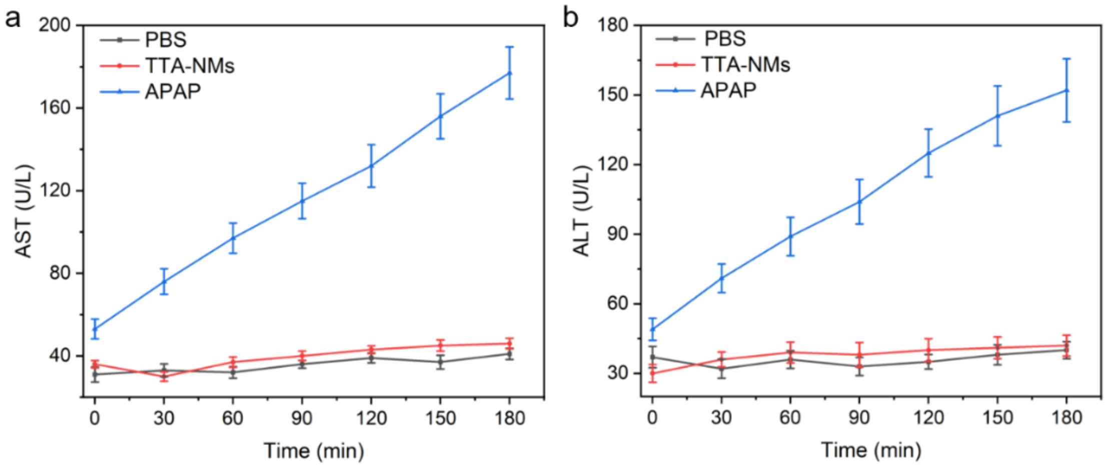

Supplementary Figure 42. Demonstration of hepatotoxicity induced by APAP treatment. Changes of AST (**a**) and ALT (**b**) contents over time toward different drugs treatment. The results are presented as mean ± standard deviations (SD) (n = 3).

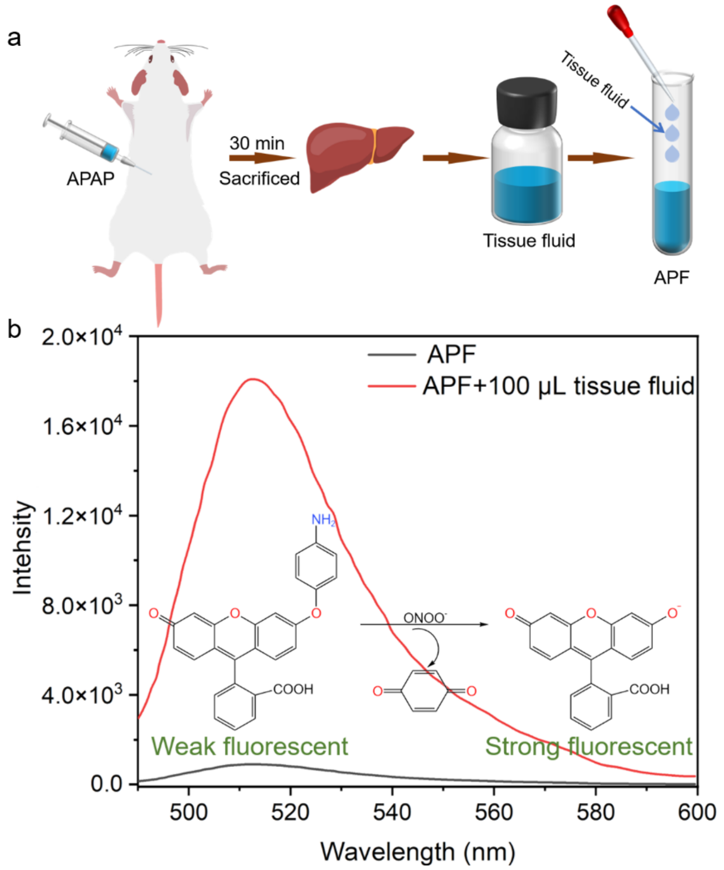

Supplementary Figure 43. Determination of the correctness of APAP-induced mouse liver toxicity model by introducing standard probes. (**a**) Schematic diagram illustrating the process to obtain liver tissue fluid of the APAP treated mouse and its addition to APF solution. Partial drawing elements were sourced from Servier Medical Art database under a Creative Commons license CC BY 3.0. (**b**) Fluorescence spectra of APF (1 mL; 10 μM) in the absence and presence of liver tissue fluid (Ex = 495 nm).

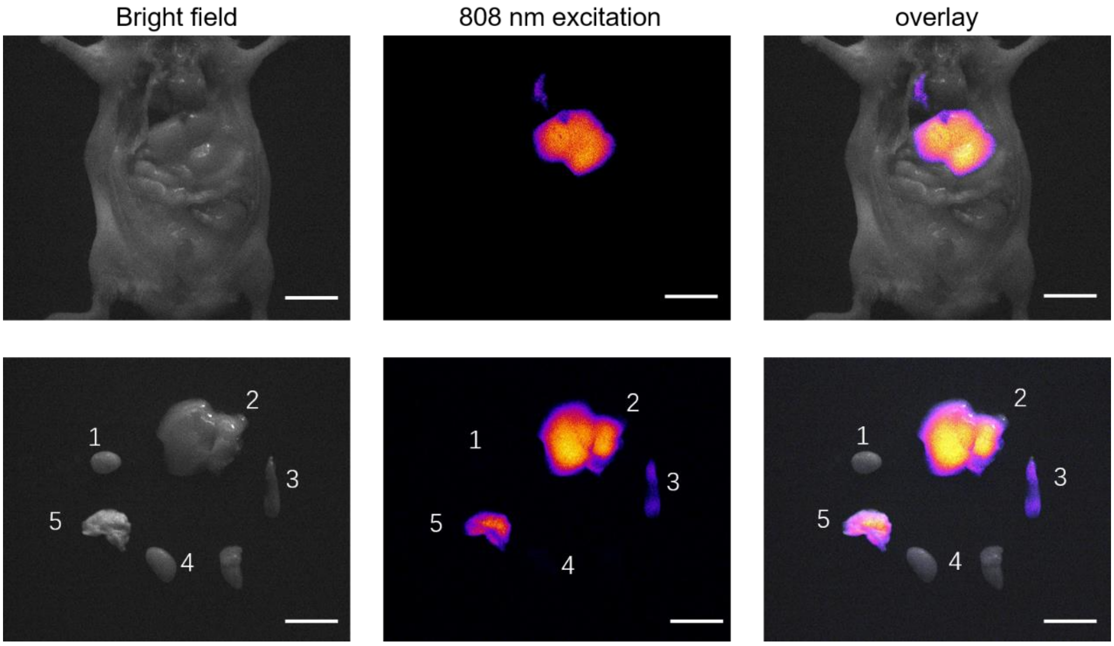

Supplementary Figure 44. Ex vivo imaging of organs harvested from mice treated with APAP-induced hepatotoxicity. Bright field (left column), TTA-UC (middle column), and overlay image (right column) are presented. The organs examined included heart (1), liver (2), spleen (3), kidney (4), and lung (5); scale bar: 1 cm.

4. Supplementary Tables

Supplementary Table 3. Crystal Data and Structure Refinement for BTTQD 1.

	BTTQD 1
Empirical formula	$C_{79}H_{95}N_8S_4Si_4$
Formula weight	1503.57
Melting point ranges	200-380 °C
Crystal system	triclinic
Space group	*P*-1
a/Å	12.1310(8)
b/Å	15.3715(9)
c/Å	23.7472(15)
α/	95.946(2)
β/	101.157(2)
γ/	104.538(2)
V/Å^3	4151.1(5)
Z	2
μ/mm^{-1}	0.314
F(000)	1592.0
Reflections collected	14589
GOF on F^2	1.051
Final R_1^a, wR_2^b[I>2σ (I)]	R_1 = 0.0688, wR_2 = 0.1824
Final R_1^a, wR_2^b (all data)	R_1 = 0.1023, wR_2 = 0.2162

$^a R_1 = \Sigma||F_o| - |Fc||/\Sigma|F_o|$. $^b wR_2 = \Sigma[w(F_o^2 - F_c^2)^2]/\Sigma[w(F_o^2)^2]^{1/2}$.

Supplementary Table 4. Electrochemical properties of BTTQD 1-3.

	S_1[a] (eV)	T_1[b] (eV)	E_{LUMO}[a] (eV)	E_{HOMO}[a] (eV)	E_{LUMO}[b] (eV)	E_{LUMO}[b] (eV)	PLQY (%)
Monomer	2.14	1.21	-3.85	-5.87	-3.62	-5.87	87
BTTQD **1**	2.07	1.10	-3.79	-5.85	-3.68	-5.88	55
BTTQD **2**	2.09	1.17	-3.78	-5.83	-3.66	-5.86	63
BTTQD **3**	2.02	1.08	-3.55	-5.41	-3.55	-5.37	51

[a]Experimental results; [b]DFT calculated results

Supplementary Table 5. Blood biochemistry and hematology data of mice.

Project name	Control group	Experiment group	reference
WBC	12.9 (*10^9/L)	13.5 (*10^9/L)	2.5~15.0
LYM#	6.64 (*10^9/L)	7.81 (*10^9/L)	0.00-15.0
MON#	6.18 (*10^9/L)	4.35 (*10^9/L)	0.00-15.0
GRA#	10.07 (*10^9/L)	5.34 (*10^9/L)	0.00-15.0
LYM%	29.0 (%)	44.6 (%)	0.0~99.9
MON%	27.0 (%)	24.9 (%)	0.0~99.9
GRA%	44.0 (%)	30.5 (%)	0.0~99.9
HGB	115.3 (g/L)	168.0 (g/L)	110.0~170.0
RBC	10.24 (*10^{12}/L)	7.22 (*10^{12}/L)	6.5~12.5
HCT	45.0 (%)	39.3 (%)	35.0~50.0
MCV	54.0 (fL)	47.2 (fL)	45.0~60.0
MCH	18.0 (pg)	18.6 (pg)	14.0~20.0
MCHC	278 (g/L)	307 (g/L)	250~350
RDWSD	24 (fL)	23 (fL)	0~99
RDWCV	13.6 (%)	12.1 (%)	0~99.9
PLT	917 (*10^9/L)	637 (*10^9/L)	500~1500
PCT	0.415 (%)	0.449 (%)	0.0~0.999
MPV	15.4 (fL)	12.2 (fL)	0~99.9
PDW	32.3 (fL)	27.5 (fL)	0~99.9
PLCR	54.5 (%)	33.0 (%)	0~99.9

Notice: White blood cells (WBC), Lymphocyte (LYM), Monocyte (MON), Granulocyte, (GRA), hemoglobin (HGB), Red blood cells (RBC), Hematocrit (HCT), Mean corpuscular volume (MCV), Mean corpuscular haemoglobin (MCH), Mean corpuscular hemoglobin concentration (MCHC), Standard deviation of red blood cell distribution width (RDWSD), Red blood cell volume distribution width (RDWCV), platelets (PLT), Procalcitonin (PCT), platelet crit (PCT), Mean platelet volume (MPV), Platelet distribution width (PDW) and Platelet-large cell ratio (PLCR).

Supplementary Table 6. Standard Deviation (σ) calculation details for the detection of $ONOO^-$ for TTA-NMs.

Number of test	UC emission intensities of TTA-NMs before $ONOO^-$ treatment	Average value	Standard deviation (σ)
1	996.7		
2	994.8		
3	997.9	996.54	1.036
4	995.1		
5	998.2		

Supplementary Table 7. Selected LOD values for $ONOO^-$ for TTA-NMs and other sensors.

Probe	Mode of sensing		Ex/Em	Response time	LOD (Ex-vivo)	Ref
tryptophan doped carbon dots	Turn - off	In vitro	370/486 nm	<100 s	1.5 µM	Anal. Chim. Acta. 2014, 852, 174
$CySO_3CF_3$ organic molecule	Turn-on	In vivo	680/710 nm	<3 min	53 nM	Anal. Chem. 2018, 90, 9301
Hf-Uio-66-$B(OH)_2$ MOF	Turn-on	Ex vivo	330/426 nm	1 min	9 nM	Inorg. Chem. 2018, 57, 16, 10128
MBTBE organic molecule	Turn-on	Ex vivo	520/569 nm	1 min	16 nM	Sens. Actuators B Chem. 2020, 303, 127284
Ru-organic complex	Turn - off	In vitro	468/600 nm	10 s	-	Spectrochim. Acta, Part A. 2012, 94, 340
TTA-NMs (Upconversion)	Turn-on	In vivo	808/690 nm	< 1 min	9 nM	This work

5. Supplementary References

1. Wang, X. Y. et al. A hybrid molecular sensitizer for triplet fusion upconversion. *Chem. Eng. J.* **426,** 131282 (2021).
2. Ai, X. et al. Multispectral optoacoustic imaging of dynamic redox correlation and pathophysiological progression utilizing upconversion nanoprobes. *Nat. Commun.* **10**, 1087 (2019).
3. Ding, F. W. et al. Indenone-fused N-heteroacenes. *Chem. Eur. J.* **25**, 15106-15111 (2019).
4. Ding, F. W. et al. Sulfur-Containing Bent N-Heteroacenes. *J. Mater. Chem. C* **7**, 14314 (2019).
5. Peng, S. et al. A cell-compatible PEO-PPO-PEO (Pluronic(R))-based hydrogel stabilized through secondary structures. *Mater. Sci. Eng. C Mater. Biol. Appl.* **69**, 421-428 (2016).
6. Li, P. H. et al. Dual responsive oligo(lysine)-modified Pluronic F127 hydrogels for drug release of 5-fluorouracil. *RSC Adv.* **10**, 24507-24514 (2020).
7. Zhang, Q. et al. Bright and stable NIR-II J-aggregated AIE dibodipy-based fluorescent probe for dynamic in vivo bioimaging. *Angew. Chem. Int. Ed.* **60**, 3967-3973 (2021).
8. Alifu, N. et al. Single-molecular near-infrared-II theranostic systems: ultrastable aggregation-induced emission nanoparticles for long-term tracing and efficient photothermal therapy. *ACS Nano* **12**, 11282-11293 (2018).
9. Peng, J. et al. Real-time in vivo hepatotoxicity monitoring through chromophore-conjugated photon-upconverting nanoprobes. *Angew. Chem. Int. Ed.* **56**, 4165-4169 (2017).
10. Zhang, J. et al. Petoud, S., Sensitization of near-infrared-emitting lanthanide cations in solution by tropolonate ligands. *Angew. Chem. Int. Ed.* **44**, 2508-2512 (2005).
11. Wang, T. et al. A hybrid erbium(III)-bacteriochlorin near-infrared probe for multiplexed biomedical imaging. *Nat. Mater.* **20**, 1571-1578 (2021).

12. Wu, Y. et al. Exceptional intersystem crossing in di(perylene bisimide)s: a structural platform toward photosensitizers for singlet oxygen generation. *J. Phys. Chem. Lett.* **1**, 2499-2502 (2010).
13. Ronchi, A. et al. High photon upconversion efficiency with hybrid triplet sensitizers by ultrafast hole-routing in electronic-doped nanocrystals. *Adv. Mater.* **32**, 2002953 (2020).
14. Cheng, Y. Y. et al. Kinetic analysis of photochemical upconversion by triplet−triplet annihilation: beyond any spin statistical limit. *J. Phys. Chem. Lett.* **1**, 1795-1799 (2010).
15. Monguzzi, A. et al. Energy transfer enhancement by oxygen perturbation of spin-forbidden electronic transitions in aromatic systems. *Phys. Rev. B* **82**, 125113 (2010).
16. Monguzzi, A. et al. Upconversion-induced fluorescence in multicomponent systems: Steady-state excitation power threshold. *Phys. Rev. B* **78**, 195112 (2008).
17. Fan, C. et al. Efficient triplet–triplet annihilation upconversion with an anti-Stokes shift of 1.08 eV achieved by chemically tuning sensitizers. *J. Am. Chem. Soc.* **141**, 15070-15077 (2019).
18. Ronchi, A. & Monguzzi, A., Sensitized triplet–triplet annihilation based photon upconversion in full organic and hybrid multicomponent systems. *Chem. Phys. Rev.* **3**, 41301 (2022).
19. Kanofsky, J. R. & Sima, P. D., Structural and environmental requirements for quenching of singlet oxygen by cyanine dyes. *Photochem. Photobiol.* **71**, 361-368 (2007).
20. Renikuntla, B. R. et al. Improved photostability and fluorescence properties through polyfluorination of a cyanine dye. *Org. Lett.* **6**, 909-912 (2004).
21. Zhang, J. et al. A dual-modal molecular probe for near-infrared fluorescence and photoacoustic imaging of peroxynitrite. *Anal. Chem.* **90**, 9301-9307 (2018).
22. Liu, R. et al. Aptamer and IR820 dual-functionalized carbon dots for targeted cancer therapy against hypoxic tumors based on an 808 nm laser-triggered three-pathway strategy. *Adv. Therap.* **1**, 1800041 (2018).
23. Uttiya, S. et al. Connecting molecule oxidation to single crystal structural and

charge transport properties in rubrene derivatives. *J. Mater. Chem. C* **2**, 4147-4155 (2014).

24. Tinker, L. A. & Bard, A. J., Electrochemistry in liquid sulfur dioxide. 1. Oxidation of thianthrene, phenothiazine, and 9,10-diphenylanthracene. *J. Am. Chem. Soc.* **101**, 2316-2319 (2002).
