# Supplementary material for: Molecular near-infrared triplet-triplet annihilation upconversion with eigen oxygen immunity
Source: Nat Commun. 2024 Mar 9;15:2157. doi: 10.1038/s41467-024-46541-z (PMC10924867; doi:10.1038/s41467-024-46541-z)
Supplement: Supplementary file 3 — Description of Additional Supplementary Files [file 41467_2024_46541_MOESM3_ESM.pdf]

Description of Additional Supplementary Files

File Name: Supplementary Data 1
Description: Optimized Cartesian coordinates for BTTQD 1-3 (from Supplementary Figures 13-15).
