# Supplementary material for: Molecular near-infrared triplet-triplet annihilation upconversion with eigen oxygen immunity
Source: Nat Commun. 2024 Mar 9;15:2157. doi: 10.1038/s41467-024-46541-z (PMC10924867; doi:10.1038/s41467-024-46541-z)
Supplement: Supplementary file 5 — Reporting Summary [file 41467_2024_46541_MOESM5_ESM.pdf]

nature portfolio

Corresponding author(s): Guanying Chen

Last updated by author(s): Feb 29, 2024

Reporting Summary

Nature Portfolio wishes to improve the reproducibility of the work that we publish. This form provides structure for consistency and transparency in reporting. For further information on Nature Portfolio policies, see our Editorial Policies and the Editorial Policy Checklist.

Statistics

For all statistical analyses, confirm that the following items are present in the figure legend, table legend, main text, or Methods section.

n/a	Confirmed	
☐	☒	The exact sample size (*n*) for each experimental group/condition, given as a discrete number and unit of measurement
☐	☒	A statement on whether measurements were taken from distinct samples or whether the same sample was measured repeatedly
☒	☐	The statistical test(s) used AND whether they are one- or two-sided<br>*Only common tests should be described solely by name; describe more complex techniques in the Methods section.*
☐	☒	A description of all covariates tested
☒	☐	A description of any assumptions or corrections, such as tests of normality and adjustment for multiple comparisons
☐	☒	A full description of the statistical parameters including central tendency (e.g. means) or other basic estimates (e.g. regression coefficient) AND variation (e.g. standard deviation) or associated estimates of uncertainty (e.g. confidence intervals)
☒	☐	For null hypothesis testing, the test statistic (e.g. *F*, *t*, *r*) with confidence intervals, effect sizes, degrees of freedom and *P* value noted<br>*Give P values as exact values whenever suitable.*
☒	☐	For Bayesian analysis, information on the choice of priors and Markov chain Monte Carlo settings
☒	☐	For hierarchical and complex designs, identification of the appropriate level for tests and full reporting of outcomes
☒	☐	Estimates of effect sizes (e.g. Cohen's *d*, Pearson's *r*), indicating how they were calculated

Our web collection on statistics for biologists contains articles on many of the points above.

Software and code

Policy information about availability of computer code

Data collection	NIR-II imaging was performed on a customized animal imaging system (In Vivo Master, Wuhan Grand-imaging Technology Co., Ltd) installed with an InGaAs camera (C-RED2, France).<br>Crystal structure was solved by using Patterson methods (SHELXS-97), expanded using Fourier methods, and refined using SHELXL-97 (full-matrix least-squares on F2) and WinGX v1.70.01 programs packages.<br>MALDI-TOF MS analyses were performed in positive reflection mode on a 5800 proteomic analyzer (Applied Biosystems, Framingham, MA, USA) with a YAG:Nd laser.<br>Dynamic light scattering (DLS) was carried out on a Malvern Zetasizer 3600 (Malvern Instruments).
Data analysis	Imaging analysis: imageJ 1.52p (NIH, USA);<br>Data representation: Origin2019, Adobe illustrator CS6;<br>NMR spectra: MestReNova 6.1.0-6224;

For manuscripts utilizing custom algorithms or software that are central to the research but not yet described in published literature, software must be made available to editors and reviewers. We strongly encourage code deposition in a community repository (e.g. GitHub). See the Nature Portfolio guidelines for submitting code & software for further information.

Data

Policy information about availability of data

All manuscripts must include a data availability statement. This statement should provide the following information, where applicable:

- Accession codes, unique identifiers, or web links for publicly available datasets
- A description of any restrictions on data availability
- For clinical datasets or third party data, please ensure that the statement adheres to our policy

The source data generated in this study have been deposited in the Figshare database under accession code https://doi.org/10.6084/m9.figshare.22976144.
The Crystallographic data for BTTQD 1 (No.2099068) reported in this study can are available in the Cambridge Crystallographic Data Center (CCDC) database under accession code www.ccdc.cam.ac.uk/data_request/cif.

Research involving human participants, their data, or biological material

Policy information about studies with human participants or human data. See also policy information about sex, gender (identity/presentation), and sexual orientation and race, ethnicity and racism.

Reporting on sex and gender	N/A
Reporting on race, ethnicity, or other socially relevant groupings	N/A
Population characteristics	N/A
Recruitment	N/A
Ethics oversight	N/A

Note that full information on the approval of the study protocol must also be provided in the manuscript.

Field-specific reporting

Please select the one below that is the best fit for your research. If you are not sure, read the appropriate sections before making your selection.

☒ Life sciences ☐ Behavioural & social sciences ☐ Ecological, evolutionary & environmental sciences

For a reference copy of the document with all sections, see nature.com/documents/nr-reporting-summary-flat.pdf

Life sciences study design

All studies must disclose on these points even when the disclosure is negative.

Sample size	Target sample size was determined by previous experience (Nature Communications. 2019, 10, 1087; 2024, 15, 170) with the methods used in this study, including biodistribution, cell viability, histological studies of hematoxylin and eosin, small animal imaging. For the in vivo and ex vivo imaging, experiment performed with 3 independent mice with similar results. No statistical methods were used to predetermine sample sizes. All of our experiments followed well-established reported protocols.
Data exclusions	No data were excluded in this study.
Replication	ROS assay in vitro experiments were repeated three times independently with similar results. Living cell staining was repeated independently three times with similar results. Cell viability assay was repeated three times independently of n = 3 with similar results. For the in vivo and ex vivo imaging, experiment was repeated three times independently of n = 3 mice with similar results. Tissue slices (n = 3) were prepared and scanned with similar results.
Randomization	Mice were randomly selected from different cages for the experiment. Different synthetic batches of nanoprobes were randomly injected into different mice. Cells were randomly pipetted into wells prior to any live-cell microscopy experiments at equivalent cell densities.
Blinding	Due to limited personnel, the researchers did not blindly assign groups in the process of experiment and data analysis.

Reporting for specific materials, systems and methods

We require information from authors about some types of materials, experimental systems and methods used in many studies. Here, indicate whether each material, system or method listed is relevant to your study. If you are not sure if a list item applies to your research, read the appropriate section before selecting a response.

Materials & experimental systems

n/a	Involved in the study
☒	☐ Antibodies
☐	☒ Eukaryotic cell lines
☒	☐ Palaeontology and archaeology
☐	☒ Animals and other organisms
☒	☐ Clinical data
☒	☐ Dual use research of concern
☒	☐ Plants

Methods

n/a	Involved in the study
☒	☐ ChIP-seq
☒	☐ Flow cytometry
☒	☐ MRI-based neuroimaging

Eukaryotic cell lines

Policy information about cell lines and Sex and Gender in Research

Cell line source(s)	HeLa cells were obtained from the Cell Bank of the Chinese Academy of Sciences.
Authentication	All cell lines were authenticated by Short Tandem Repeat test.
Mycoplasma contamination	All cell lines were tested negative for mycoplasma contamination.
Commonly misidentified lines (See ICLAC register)	The HeLa cell line is typically difficult to misidentify as it serves as a source of contamination for many misidentified cell lines. Additionally, we did not detect any mycoplasma contamination from it.

Animals and other research organisms

Policy information about studies involving animals; ARRIVE guidelines recommended for reporting animal research, and Sex and Gender in Research

Laboratory animals	The 4-week-old female Kunming mice were purchased from the Second Affiliated Hospital of Harbin Medical University. The mice were then housed in regular rectangular cages and maintained under standard conditions in conventional housing facilities (light/dark cycle: 16/8 hours; Temp: 25 degree centigrade℃; relative humidity: 40%).
Wild animals	The study did not involve the use of wild animals.
Reporting on sex	There was no sexual activity involved in the study.
Field-collected samples	The study did not involve samples collected from the field.
Ethics oversight	All animal experiments are conducted in accordance with the Institutional Animal Care and approved by the Ethics Committee of Harbin Institute of Technology.

Note that full information on the approval of the study protocol must also be provided in the manuscript.

Plants

Seed stocks	N/A
Novel plant genotypes	N/A
Authentication	N/A
